# In Search of Glacial Refuges of the Land Snail *Orcula dolium* (Pulmonata, Orculidae) - An Integrative Approach Using DNA Sequence and Fossil Data

**DOI:** 10.1371/journal.pone.0096012

**Published:** 2014-05-07

**Authors:** Josef Harl, Michael Duda, Luise Kruckenhauser, Helmut Sattmann, Elisabeth Haring

**Affiliations:** 1 Central Research Laboratories, Museum of Natural History Vienna, Vienna, Austria; 2 Department of Integrative Zoology, University of Vienna, Vienna, Austria; 3 Department of Invertebrate Zoology, Museum of Natural History Vienna, Vienna, Austria; Australian Museum, Australia

## Abstract

Harboring a large number of endemic species, the Alps and the Western Carpathians are considered as major centers of biodiversity. Nonetheless, the general opinion until the turn of the millennium was that both Central European mountain regions did not provide suitable habitat during the Last Glacial Maximum, but were colonized later from southern refuges. However, recent molecular genetic studies provide new evidence for peripheral Alpine refuges. We studied the phylogeography of the calciphilous land snail *O. dolium* across its distribution in the Alps and the Western Carpathians to assess the amount of intraspecific differentiation and to detect potential glacial refuges. A partial sequence of the mitochondrial COI was analyzed in 373 specimens from 135 sampling sites, and for a subset of individuals, partial sequences of the mitochondrial 16S and the nuclear histone H3 and H4 were sequenced. A molecular clock analysis was combined with a reconstruction of the species’ geographic range history to estimate how its lineages spread in the course of time. In order to obtain further information on the species’ past distribution, we also screened its extensive Pleistocene fossil record. The reconstruction of geographic range history suggests that *O. dolium* is of Western Carpathian origin and diversified already around the Miocene-Pliocene boundary. The fossil record supports the species’ presence at more than 40 sites during the last glacial and earlier cold periods, most of them in the Western Carpathians and the Pannonian Basin. The populations of *O. dolium* display a high genetic diversity with maximum intraspecific *p*-distances of 18.4% (COI) and 14.4% (16S). The existence of various diverged clades suggests the survival in several geographically separated refuges. Moreover, the sequence patterns provide evidence of multiple migrations between the Alps and the Western Carpathians. The results indicate that the Southern Calcareous Alps were probably colonized only during the Holocene.

## Introduction

The Pleistocene climate changes shaped the phylogeographic patterns of various organisms [1]. In particular, the severe cooling starting with the end of the Early Pleistocene (about 900 kya) was the starting point for massive glaciations in the northern hemisphere [2]. Mountainous regions such as the Central European Alps were heavily affected due to shifts in temperature and humidity, and the expansion of glaciers, resulting for many taxa in the fragmentation of populations, complete or local extinction, and the loss of variation due to genetic bottlenecks [1]. Thereby, the Last Glacial Maximum (LGM; 30–18 kya [3]) is of most relevance in respect to the current distribution of Central European species. Although the existence of glacial refuges at the periphery of the Alps was discussed more than half a century ago [4], the general opinion until end of the 20th century was that glacial refuges were located mainly in southern regions [1]. However, molecular genetic analyses and fossil data revealed the existence of northern refuges in the Western Carpathians [5], [6] and the Pannonian Basin [7]. Several peripheral Alpine refuges were proposed for silicophilous mountain plants [8] and calciphilous land snails such as *Arianta arbustorum* [9]–[12], *Carychium minimum*, *Carychium tridentatum* [13], *Trochulus oreinos* [14] and *Trochulus villosus* [15].

Most molecular genetic studies investigating glacial refuges were based on the assumption that populations diverged during isolation in geographically separated areas, and that populations of former refuge areas are now characterized by high genetic diversity and the presence of rare (private) alleles [16]. Nevertheless, a major obstacle in identifying signatures of Pleistocene refuges is that phylogenetic signals are blurred because of migration and intermixture of previously separated populations. Therefore, species showing low dispersal capabilities and specific habitat requirements, which is the case in most land snails, might be suited to infer past distributional patterns.

In the present study, we investigate the phylogeographic patterns of the land snail *Orcula dolium* (Draparnaud, 1801). The species inhabits all major limestone areas of the Alps and the neighboring Western Carpathians [17], [18]. *O. dolium* is usually associated with mountainous forest habitats or rocky landscapes with patches of vegetation, but it is also found on rocky slopes at high altitudes up to 2160 m above sea level (asl) [17] (and data of present study). Within the Alps, the east-west orientated Central Alps, consisting mainly of silicate rock, represent the largest distributional barrier for the species; they separate the Northern Calcareous Alps from the Southern Calcareous Alps. The Vienna Basin, closing the Pannonian Basin to the north, constitutes another distributional gap because it separates the populations of the Northern Calcareous Alps (including the Wienerwald) and the Western Carpathians. Recent investigations of loess profiles from the Pannonian Basin show that during the Late Pleistocene *O. dolium* also occurred in the lowlands of the region [19], although the species seems to have vanished from it during the Holocene. The margins of the Northern Calcareous Alps and the Western Carpathians, enclosing the Vienna Basin east and west, harbor the majority of the described 23 subspecies [20]. As these areas were only partially glaciated during the LGM, they also come into consideration as potential refuges for the species.

We perform a comprehensive phylogeographic study of *O. dolium* analyzing mt and nc markers to detect potential glacial refuges and to assess the amount of intraspecific variability. Furthermore, we test whether the described subspecies are differentiated genetically in the mitochondrial (mt) and nuclear (nc) markers. Our sample covers almost the entire range of the species. We include data of the Pleistocene fossil record to obtain insights into the species’ past distribution. In order to estimate which potential areas were inhabited by ancestral populations of *O. dolium* and to trace the distribution patterns of the mt lineages throughout time, a molecular clock analysis is performed and combined with a phylogeographic range reconstruction.

## Methods

### Study Area and Sampling

**Figure 1:**
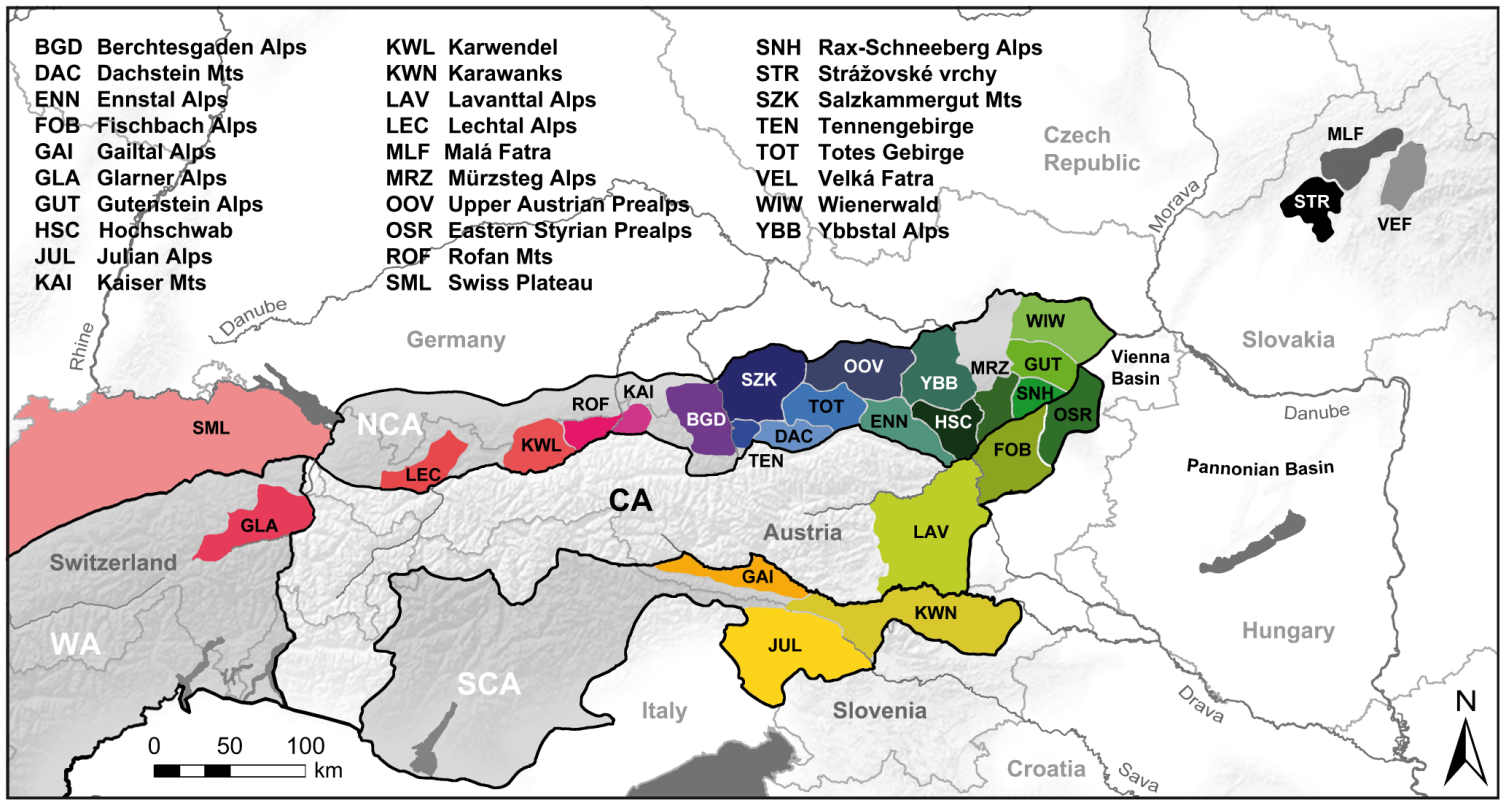
Distribution of mountain areas investigated in the present study. The colors correspond to those used in the mt trees (Figs. 1 and 4) and in the histone H4/H3 network (Fig. 5). The outlines of the Northern Calcareous Alps (NCA), the Southern Calcareous Alps (SCA), the Western Alps (WA) and the Central Alps (CA) are framed in black. The names of the mountain regions and abbreviations are provided in the figure.

Specimens were collected from a large part of the species’ distribution, including several Alpine and Western Carpathian type localities. *O. dolium* is not protected by conservation laws of the countries where the collections were performed. Thus, in general, permissions were not necessary. For protected areas in Austria, permissions were provided by federal states authorities. Permit numbers: RU5-BE-64/011-2013 (Lower Austria), FA13C-53 Sch 6/6–2007 (Styria) and N10-117-2008 (Upper Austria). Most samples of *O. dolium* were collected in the Northern Calcareous Alps (Austria and Germany), a lesser fraction in the Western Carpathians (Slovakia), the Southern Calcareous Alps (Austria, Slovenia and Italy) and the Western Alps (Switzerland), totaling 373 specimens of 135 sites (Table 1). The habitats include wooded areas in the lowland, mountainous vegetated areas and rocky slopes in the alpine zones, with an altitudinal range from 120 m to 2160 m asl. Elevation and position were determined via GPS. At every sample locality, if available, a minimum of three living specimens was collected, prepared for DNA analyses and stored in 80% ethanol following the protocol of [21]. *Orcula conica* (Rossmässler, 1837) (ID: 3899; Trögener Klamm, Carinthia) was used as outgroup. Selected type specimens of *O. dolium* subspecies included in the present study are shown in Fig. S1. For the inference of substitution rates used in the molecular clock analysis, six specimens of *Orculella bulgarica* (Hesse, 1915) and four specimens of *Orculella aragonica* (Westerlund, 1897) were included. Samples of *Orculella bulgarica* were collected by Barna Páll-Gergely (Shinshu University, Matsumoto, Japan; Turkish samples) and Alexander Reischuetz (Greek samples). DNA samples of *O. aragonica* were obtained from Benjamín Gómez-Moliner (Universidad del País Vasco, Vitoria, Spain). Voucher specimens of the first three taxa are deposited in the Natural History Museum Vienna, the whereabouts of the *O. aragonica* vouchers are provided in [22]. Every individual sample was assigned an ID consisting of a unique specimen number and a locality tag. The latter encodes the Alpine geographic mountain region as classified in the SOIUSA system [23], with localities of each region numbered from west to east (Table 1). Due to their geographic vicinity to the Northern Calcareous Alps, the sites located in the Fischbach Alps, Eastern Styrian Prealps and Lavanttal Alps as well as those of the Wienerwald, which are classified to the Central Alps in the SOIUSA system, are treated as Northern Calcareous Alpine mountain regions here. A map providing an overview of the mountain regions investigated is shown in Fig. 1. The Slovakian sites were each assigned to one of the geological areas defined for the Carpathians [24]. To illustrate distribution patterns, the haplotypes in the phylogenetic trees and the histone network are marked by different colors, corresponding to the SOIUSA mountain regions as shown in Fig. 1.

**Table 1:** List of localities and individuals included in the present study.

locality code	Locality	N	E	m. asl	clade(mt)	histonealleles	IndID	Sub-species
**Western Carpathians**								
MLF1	SK, TC, PovazskáBystrica, Považský hrad	49°8.734′	18°27.422′	500	**2, 3, 9**	**HT1 var**	3937 (2); 3936, 3938, 3940,3941, 3943 (3); 3939, 3942 (9);	*d*
MLF2	SK, TC, PovazskáBystrica, Manínskatiesňava	49°8.398′	18°30.421′	380	**1**	**HT3**	3918, 3919, 3920, 3921,3922, 3923, 3924, 3925	b *
MLF3	SK, TC, PovazskáBystrica, Manínskatiesňava	49°8.366′	18°30.475′	400	**1**	**HT3** **var/HTX**	3912, 3913, 3914, 3915,3917	b *
MLF4	SK, ZI, Súľov-Hradná,Súľovské skaly	49°10.101′	18°34.633′	315	**1, 9**	**HT3**	3930, 3931, 3932, 3934,3935 (1); 3933 (9);	*d*
MLF5	SK, ZI, Malá Fatra,Fackov	49°0.007′	18°35.853′	556	**7, 10**	**HTX**	1374 (7); 1375, 1376, 1380 (10);	*d*
MLF6	SK, ZI, RajeckéTeplice, SkalkyStrážovské	49°8.115′	18°41.745′	470	**1, 3**	**HT3/HTX**	3952, 3953, 3954 (1); 3944,3945, 3946, 3947, 3948, 3949,3950, 3951 (3);	m *
MLF7	SK, ZI, Malá Fatra,Terchová-Vrata	49°14.664′	19°2.36′	564	**7**	**HTX**	1372, 1373	*d*
STR1	SK, TC, TrenčianskeTeplice, MalýKlepáč	48°53.72′	18°10.649′	480	**9**	**HT1**	3908, 3909, 3910	t *
STR2	SK, TC, Strážovskévrchy, ValaskaBeta	48°53.519′	18°22.469′	445	**9**	**HTX**	1996	*d*
VEF1	SK, ZI, Ružomberok,Cebrat (S side)	49°5.474′	19°17.174′	700	**5**	**HT3**	3926, 3927, 3928, 3929	***c***
**Northern Calcareous Alps**								
BGD1	DE, BY, Ruhpolding,Brand	47°45.004′	12°37.92′	681	**1**		4088, 4089, 4090	*d*
BGD2	DE, BY, BadReichenhall,Obernesselgraben	47°42.593′	12°48.569′	605	**1**		4086, 4087	*d*
BGD3	AT, S, Steinernes Meer,Oberweissbachgraben(mid)	47°28.288′	12°51.716′	740	**8**		4113, 4114, 4115	*d*
BGD4	AT, S, Steinernes Meer,Einsiedler	47°26.622′	12°51.72′	1029	**8**	**HT2**	4117, 4118, 4119	*d/e/r*
BGD5	AT, S, Steinernes Meer,Oberweissbachgraben(E end)	47°28.298′	12°51.997′	850	**8**		4109, 4110, 4111	*d/e/r*
BGD6	AT, S, Steinernes Meer,Wiechenthalerhütte(ascent)	47°27.652′	12°52.122′	1413	**8**		4121	*d/e/r*
BGD7	DE, BY, Wimbachtal,Mittleres Wimbachtal	47°34.596′	12°54.064′	1000	**1**		4083, 4084, 4085	*d/e/r*
BGD8	AT, S, Untersberg, Fürstenbrunn	47°44.523′	12°59.582′	515	**1**		4126, 4127, 4128	*d*
BGD9	AT, S, Untersberg,Untersberg (N side)	47°43.703′	13°0.497′	1530	**1**		4122, 4123	*d/e/r*
BGD10	DE, BY, Untersberg,Eckberg	47°41.312′	13°1.089′	747	**1**		4079, 4080, 4081	*d*
BGD11	AT, S, Hochkönig,Dientner Sattel (Elmau)	47°23.713′	13°4.924′	1256	**1, 8**	**HT1 var**	1173 (1); 1172 (8);	*d*
DAC1	AT, OOE, Dachstein,Klausbrunn	47°32.787′	13°36.755′	940	**1**		1357, 1377, 1378	*d*
DAC2	AT, S, Salzberg, Hallstatt(Plassenwanderweg 1177)	47°34.151′	13°37.482′	1177	**1**		1125	*d*
DAC3	AT, OOE, Dachstein,Wiesbergalm(Wiesberghaus)	47°31.529′	13°37.493′	1685	**8**	**HT1 var**	1279, 1280, 1281	*d*
DAC4	AT, ST, Grimming,Grimming (SE ascent 2)	47°31.172′	14°2.326′	1149	**8**	**HT2**	5641, 5642, 5643	*d*
DAC5	AT, ST, Grimming,Grimminghütte	47°30.865′	14°2.962′	938	**8**		5638, 5639, 5640	*d*
ENN1	AT, ST, Haller Mauern,Groβer Pyhrgas(Hiaslalm)	47°38.638′	14°22.595′	1292	**1**		3083, 3084, 3085	*d*
ENN2	AT, ST, Johnsbachtal,Langriesgraben	47°33.641′	14°34.64′	704	**1**		1135, 1136, 1137	*d*
ENN3	AT, ST, Johnsbachtal,Langries bridge	47°33.681′	14°34.845′	631	**1**		5646, 5647, 5648	*d*
ENN4	AT, ST, Johnsbachtal,Kaderalpl	47°34.063′	14°34.899′	634	**1**		874, 875, 876	*d*
ENN6	AT, ST, Johnsbachtal,Im Gseng 1 (below)	47°34.044′	14°34.934′	605	**1**		5654	*d*
ENN8	AT, ST, Johnsbachtal,Hellichter Stein	47°34.544′	14°35.348′	606	**1**		913, 914, 916, 918	*d*
ENN9	AT, ST, Johnsbachtal,Im Gseng 3 (mid part)	47°34.085′	14°35.687′	766	**1**		5618	*d*
ENN10	AT, ST, Johnsbachtal,Im Gseng 4	47°34.033′	14°36.106′	1039	**1**		5623, 5624, 5625	*d/e/r*
ENN11	AT, ST, Haindlkar,Zigeuner	47°35.114′	14°36.695′	603	**1**		893, 894, 895	*d*
ENN12	AT, ST, GroβesHaindlkar,Haindlkar-Hütte 2	47°34.039′	14°36.773′	1078	**1**		5632, 5633, 5634	*d*
ENN13	AT, ST, Gstatterboden,Ameishütte	47°35.744′	14°38.25′	629	**1**		1128, 1129	*d*
ENN14	AT, ST, Gstatterboden,Ameishütte	47°35.744′	14°38.25′	629	**1**		841, 842	*d*
ENN15	AT, ST, Gstatterboden,Planspitzgraben	47°35.297′	14°38.327′	668	**1**		897, 910, 911	*d*
ENN16	AT, ST, Johnsbachtal,Schröckalm	47°32.072′	14°40.051′	1377	**1**		1166, 1167, 1168	*d/e/r*
ENN17	AT, ST,Tamischbachturm,ridge on summit	47°36.932′	14°41.76′	1940	**1**		3887, 3889, 3890	*d/e/r*
ENN18	AT, ST, Groβreifling,Groβreifling	47°39.481′	14°42.669′	450	**1**		1158, 1159, 1160	*d*
ENN19	AT, ST, Hieflau,Schneckensafari	47°36.013′	14°44.778′	523	**1**		3106, 3107, 3108	*d*
FOB1	AT, ST, Schöckl,Teufelstein	47°12.525′	15°27.665′	950	**1**	**HT1**	858, 859, 860	*p*
FOB2	AT, NOE, Semmering,Roter Berg(Kalte Rinne)	47°39.411′	15°47.694′	822	**1**		629, 630, 837	*d/g/p*
FOB3	AT, NOE, Semmering,Sonnwendstein(Almweg 1229)	47°37.604′	15°51.141′	1229	**1**	**HT1/HT1** **var**	370, 371, 372	*d/g/p/e/r*
FOB4	AT, NOE, Semmering,Sonnwendstein(Almweg 1343)	47°37.682′	15°51.228′	1343	**1**		606, 1121, 1122	*d/g/p/e/r*
FOB5	AT, NOE, Semmering,Sonnwendsteinsummit	47°37.783′	15°51.62′	1523	**1**		339	*d/g/p/e/r*
FOB6	AT, NOE, Semmering,Sonnwendstein(Pollereshütte)	47°37.751′	15°51.708′	1477	**1**		361, 367	*d/g/p/e/r*
FOB8	AT, NOE, Semmering,Mariaschutz(Marterl)	47°38.276′	15°52.63′	788	**1**		602, 603, 604, 1984	*d/g/p*
GUT1	AT, NOE, Tiefental,Ochbauer	47°52.638′	15°38.854′	739	**6**	**HTX**	3041, 3042, 3043	*d*
GUT2	AT, NOE, Halbachtal,Rossbachklamm 1	47°54.327′	15°40.937′	649	**6**		3074, 3075, 3076	*d*
GUT3	AT, NOE, Halbachtal,Kleinzell	47°56.902′	15°42.874′	507	**6**		3066, 3067, 3068	*d*
GUT4	AT, NOE, Gösing,Sieding(foothill Gösing)	47°44.399′	15°58.723′	356	**1**		5471	*p*
GUT5	AT, NOE, Gösing,Gösing(foothill W side)	47°44.431′	15°59.198′	700	**1**		5472, 5473, 5474, 5475	*p*
GUT6	AT, NOE, Gösing,Gösing (W side)	47°44.401′	15°59.205′	864	**1**		2942, 2943, 2945	*d/p/e/r*
GUT7	AT, NOE, Gösing,Flatzerwand (Gösing)	47°44.829′	16°0.215′	671	**1**		2934, 2935, 2936	*p*
GUT8	AT, NOE, HoheWand, Grafenberg(Seiser Toni)	47°48.360′	16°0.503′	780	**6**	**HT1**	298, 299, 1134	*d*
HSC1	AT, ST, Hochschwab, Aflenzer Staritzen	47°38.279′	15°16.095′	1779	**1**		1358, 1392	*d/e/r*
HSC2	AT, ST, Hochschwab,Salzatal(Weichselboden)	47°39.96′	15°9.812′	660	**1**		3078, 3079, 3081	*d/e/r*
KAI1	AT, T, Wilder Kaiser,Kaiserkopf	47°33.225′	12°18.223′	1549	**1**		1138, 1140, 1982	*d*
KAI2	AT, T, Wilder Kaiser,Rote Rinne	47°33.624′	12°18.238′	2159	**1**		1119, 1120	*d*
KAI3	AT, T, Wilder Kaiser,Gamsanger	47°33.682′	12°18.428′	1938	**1**		838, 1107, 6506	*d*
KAI4	AT, T, Wilder Kaiser,Hochgrubach	47°33.357′	12°18.604′	1666	**1**	**HT1**	1142, 1143, 1981	*d*
KWL1	AT, T, Pertisau,Achensee W shore	47°27.423′	11°42.081′	955	**1**	**HT1**	614, 615, 617, 1983	*d*
LAV1	AT, ST, Gleinalpe, St.Pankrazen(Stübinggraben)	47°9.466′	15°10.934′	730	**1**		864, 865, 866	*p*
LEC1	AT, T, Imst,Hahntennjoch	47°17.225′	10°36.566′	1482	**1**	**HT1**	5932	*d*
MRZ1	AT, NOE,Göller, Gscheid	47°48.622′	15°27.084′	914	**1**		2976, 2977, 2978	*d/e/r*
MRZ2	AT, NOE,Göller, Lahnsattel	47°46.493′	15°29.145′	1020	**1**		2972, 2973, 2974	*d/e/r*
MRZ3	AT, NOE, Göller, Turmmauer	47°48.705′	15°31.118′	812	**1**		3028, 3030	*d/e/r*
MRZ4	AT, NOE, Göller, Klopfermauer	47°48.705′	15°32.264′	741	**1**		2961, 2962, 2963, 2964	*d*
MRZ5	AT, NOE,Göller, Klopfermauerwaterfall	47°48.705′	15°32.264′	741	**1**		2970, 2971	*d*
MRZ6	AT, ST, Hohe Veitsch,Wildkamm 2	47°39.676′	15°23.437′	1421	**1**		6149, 6150	*d/e/r*
MRZ7	AT, ST, Hohe Veitsch,Wildkamm 1	47°39.396′	15°24.264′	1587	**1**		6146, 6147	*d/e/r*
OOV2	AT, OOE, Traunstein,Gmundner-Hütte	47°52.323′	13°50.016′	1604	**8**	**HT2**	599, 600, 601	*d/e/r*
OOV3	AT, OOE, Traunstein,Mairalmsteig	47°51.96′	13°50.047′	1166	**8**		2810, 2811	*d/e/r*
OSR1	AT, NOE, Grimmenstein,Burgruine Grimmenstein	47°37.949′	16°7.289′	677	**1**	**HT1 var**	5611, 5613	*p*
OSR3	AT, NOE, Seebenstein,Türkensturz	47°40.876′	16°8.277′	550	**1**	**HT1**	5606, 5607, 5608	p *
ROF1	AT, T, Hochiss,Mauritz-Hochleger	47°26.629′	11°45.91′	1832	**1**		620, 621, 622	*d*
SNH11	AT, ST, Schneealpe, Kampl (Kutatschhütte)	47°41.096′	15°36.182′	1673	**1**		3328, 3329, 3330	*d/e/r*
SNH12	AT, ST, Schneealpe,Kampl (Kohlebnerstand)	47°40.871′	15°36.677′	1487	**1**		3324, 3325, 3327	*d/e/r*
SNH13	AT, ST, Schneealpe,Schauerkogel(Schneealpenhaus)	47°41.875′	15°36.696′	1742	**1**		3332, 3333, 3334	*d/e/r*
SNH14	AT, NOE, Rax, PreinerGscheid (Waldbach 1)	47°39.395′	15°41.097′	817	**1**		2947, 2948	*d/e/* r *
SNH15	AT, NOE, Rax, PreinerGscheid (Waldbach 2)	47°39.395′	15°41.097′	817	**1**		2950	*d/e/* r *
SNH16	AT, NOE, Rax, PreinerGscheid	47°40.981′	15°43.124′	1249	**1**		1123	*d/e/* r *
SNH17	AT, NOE, Rax,Seehütte	47°42.173′	15°43.33′	1761	**1**	**HT1 var**	315, 316, 317	*d/e/* r *
SNH18	AT, NOE, Rax, Göbl-Kühnsteig 1	47°41.42′	15°43.399′	1397	**1**		1111	*d/e/* r *
SNH19	AT, NOE, Höllental,Weichtalklamm	47°44.883′	15°46.249′	592	**1**		349, 350, 351	*d*
SNH20	AT, NOE, Rax,Gsolhirn-Steig	47°43.141′	15°46.993′	1400	**1**		3859, 3860, 3862	*d/e/* r *
SNH21	AT, NOE, Schneeberg,Fadenwände 1525	47°47.253′	15°48.655′	1525	**6**	**HT1 var**	1362	***e*** /r *
SNH22	AT, NOE, Schneeberg,Schneidergraben	47°46.096′	15°49.99′	1401	**1**	**HT1 var**	1387, 1389	***e*** /r *
SNH23	AT, NOE, Semmering,Adlitzgraben(Breitenstein)	47°39.361′	15°50.168′	650	**1**		395, 396, 397, 2814, 2815	***g*** /p *
SZK1	AT, S, Vordertrattbach,Lungauer Kurve	47°38.284′	13°15.425′	1295	**1**		1506, 1507	*d*
SZK2	AT, S, Seewaldtal,Seewaldsee	47°37.757′	13°16.244′	1115	**1**	**HT1 var**	1518, 1519, 1520	*d*
SZK3	AT, S, Trattberg,Christlalm	47°39′	13°16.7′	1441	**1**		1508	*d*
SZK4	AT, S, Trattberg,Parkplatz	47°38.352′	13°16.821′	1551	**1**		1501, 1502, 1503	*d*
SZK5	AT, OOE, Hochlecken,Aurach Ursprung	47°50.308′	13°37.411′	857	**8**		288, 292	*d/e/r*
SZK6	AT, OOE, Höllenkogel,Riederhütte	47°48.238′	13°40.602′	1757	**8**		636, 637, 831	*d*
SZK7	AT, OOE, Höllenkogel,Totengrabengupf	47°48.272′	13°41.192′	1736	**8**		607, 609	*d/e/r*
SZK8	AT, OOE, Hochschneid,Hochschneid	47°48.458′	13°41.533′	1624	**8**	**HT1**	346, 347, 348	*d*
SZK9	AT, OOE, Steinkogel,Groβer Steinkogel	47°48.737′	13°42.732′	1531	**8**		648, 649, 650	*d*
SZK10	AT, OOE, Feuerkogel,Pledialm	47°48.984′	13°43.549′	1444	**8**		644, 1145, 1146	*d/e/r*
TEN1	AT, S, Hochthron,Unteres Throntal	47°29.461′	13°14.091′	1717	**8**	**HT1 var**	1488, 1489, 1490	*d/e/r*
TEN2	AT, S, Hochthron,Thronleiter	47°29.31′	13°14.611′	1940	**8**	**HT1 var**	1523, 1524	*d/e/r*
TEN3	AT, S, Hochthron,Werfenerhütte	47°29.311′	13°14.611′	1969	**1, 8**	**HT1 var**	1495 (1); 1493, 1494 (8);	*d/e/r*
TOT1	AT, OOE, Groβer Priel,Vorderer Ackergraben	47°43.724′	14°2.504′	1027	**8**		3863, 3864, 3865	*d*
TOT2	AT, OOE, Groβer Priel,Mittlerer Ackergraben	47°43.48′	14°2.69′	1495	**8**		3879, 3880, 3881	*d*
TOT3	AT, OOE, Groβer Priel,Welser-Hütte	47°43.493′	14°2.99′	1747	**8**	**HT2**	3871, 3872, 3873	*d*
WIW2	AT, NOE, St. Andrä-Wördern,Hagenbachklamm	48°18.66′	16°12.582′	191	**4B**	**HT1 var/** **HT2 var/** **HTX**	385, 386, 387, 392, 393, 394	*i*
WIW3	AT, W, Mauerbach,Mauerbachtal	48°13.743′	16°12.796′	205	**4B**	**HT2**	1161, 1162	*i*
WIW8	AT, NOE, Greifenstein,Kaiserdenkmal	48°20.615′	16°15.205′	253	**4B**	**HT2**	628	*i*
YBB1	AT, NOE, Dürrenstein,Herdenglhöhle(Herdengl)	47°50.674′	14°59.006′	850	**1**		1148, 1149	*d/e/r*
YBB2	AT, NOE, Dürrenstein,Lechnergraben(Wildgatter)	47°49.94′	15°1.589′	674	**1**		1163, 1164, 1165	*d*
YBB3	AT, NOE, Dürrenstein,Wiesenalm(Hühnerkogel)	47°48.862′	15°2.034′	1475	**1**		1126, 1127	*e/r*
YBB4	AT, NOE, Dürrenstein,Am Hohen Hirzeck(Hühnerkogel)	47°48.987′	15°2.11′	1410	**1**		313, 834, 835	*e/r*
YBB5	AT, NOE, Dürrenstein,Rosseck (Legsteinquelle)	47°48.045′	15°2.523′	1480	**1**		96, 97, 98, 306, 308	*e/r*
YBB6	AT, NOE, Dürrenstein,Springkogel (Dolinenrand)	47°47.561′	15°2.803′	1665	**1**		92, 93, 94	*e/r*
YBB7	AT, NOE, Dürrenstein,Lechnergraben (Talschluss)	47°49.387′	15°2.673′	1160	**1**		300	*e/r*
YBB8	AT, NOE, Dürrenstein,Dürrenstein (ascent E side)	47°47.494′	15°4.102′	1631	**1**		95, 1124	*e/r*
YBB9	AT, ST, Kräuterin,Dürradmer	47°43.231′	15°9.64′	1100	**1**		1152	*d*
YBB10	AT, NOE, Ötscher,Hüttenkogel	47°51.297′	15°11.171′	1605	**1**		3883, 3884	*d/e/r*
YBB11	AT, NOE, Hochbärneck,Reifgräben	47°56.98′	15°11.83′	420	**1**		1933	*d*
YBB12	AT, NOE, Hochbärneck,Hochbärneck	47°55.047′	15°12.032′	850	**1**		1934, 1935, 1936	*d/e/r*
**Southern Calcareous Alps**								
GAI1	AT, K, Kreuzen, Meierle	46°41.028′	13°26.015′	927	**1**	**HT1**	1131, 1132, 1133	*d*
GAI2	AT, K, Kreuzen,Gailwaldbachgraben	46°40′	13°34.006′	880	**6**		1108, 1109	*d*
GAI3	AT, K, Kreuzen,Gailwaldbachgraben	46°40.149′	13°34.28′	880	**6**		1150, 1151	*d*
GAI4	AT, K, Dobratsch,Höhenrain	46°35.048′	13°41.041′	1900	**6**	**HT1**	640, 642	*d/e/r*
JUL1	IT, Bovec, Mt. Predil,Predil pass	46°25.148′	13°34.59′	1153	**6**		3461	*d*
JUL2	SI, Bovec, Koritnicavalley, Trdnjava Kluže 2	46°21.61′	13°35.62′	534	**6**		1944, 1945	*d*
JUL3	SI, Bovec, Koritnicavalley, Trdnjava Kluže 1	46°21.61′	13°35.62′	534	**6**		3465	*d*
JUL4	SI, Bovec, Mt. Triglav,Kluže	46°20.8′	13°30.9′	870	**6**	**HT1**	1363, 1364, 1365	*d*
JUL5	IT, FVG, Fusine inValromana, Mangart	46°27.496′	13°41.12′	1379	**6**		1509, 1510, 1511	*d*
KWN1	AT, K, Koschuta,Trögener Klamm	46°27.212′	14°29.811′	762	**6**	**HT1**	3903, 3905, 3906	*d*
**Western Alps**								
GLA1	CH, SG, Calfeisental,St. Martin	46°55.353′	9°21.333′	1347	**4A**	**HT1/HT1** **var**	5933, 5934, 6140	*d*
SML11	CH, BRN, Rumisberg,Schore (N of)	47°16,671′	7°38,210′	1066	**4**		6140	*d*
SML4	CH, BRN, Moutier,Gorges de Court	47°15.36′	7°20.61′	650	**4A**	**HT1**	6144, 6145	*d*
**Outgroup taxa**								
***Orcula conica***								
KWN1	A, K, Koschuta, TrögenerKlamm	46°27.212′	14°29.811′	762			3899	
***Orculella aragonica***								
BAE1	ES, Andalucia, Granada,Graena	31°18.44′	−3°14.81′	940			7132 (P3)	
BAE2	ES, Andalucia, Granada,Tocón	31°12,47′	−3°21.63′	1431			7133 (P6)	
BAE3	ES, Andalucia, Granada,El Baico	37°31.95′	−2°44.38′	1431			7135 (P7)	
BAE4	ES, Andalucia, Granada,Tocón	37°15.16′	−3°23,67′	1365			7137 (P9)	
***Orculella bulgarica***								
PIN1	GR, West Macedonia,Florina	40°44.1′	21°40.7′	580			7002, 7003, 7004	
PON1	TR, Erzurum, Aşkale	39°50.4′	40°33.9′	1880			6606, 6607	
PON2	TR, Erzurum, Aşkale	39°56.5′	40°36.4′	1652			7105	

The first column indicates the mountain chain: Northern Calcareous Alps (NCA), Southern Calcareous Alps (SCA), Western Alps (WA) and Western Carpathians (CAR). The SOIUSA codes each correspond to a mountain region following [23] (see Fig. 1 for full names). Country names are abbreviated according to the ISO 3166-1 code as defined by the International Organization for Standardization. The GPS coordinates are given according to the World Geodetic System 1984 (WGS84) alongside the altitude in meters above sea level (asl). The individual IDs (IndIDs) of the specimens, together with information on the respective mt clades, are provided for each locality. The attachment ‘var’ indicates that specimens provided variants slightly deviating from the three main alleles, and ‘HTX’ are strongly differing unique alleles. The last column indicates the subspecies reported by [17] for the respective Alpine areas (*e: edita, r: raxae, p: pseudogularis, i: infima, d: dolium, g: gracilior*) or represent type localities of Carpathian subspecies (*b: brancsikii, c: cebratica, m: minima, t: titan*). Asterisks indicate that the sampling site is the type locality of the respective subspecies. Abbreviations of country names and federal districts are provided at the end of the table.

### Molecular Markers and Primer Design

DNA was extracted from 373 *O. dolium* specimens with the QIAgen Blood and Tissue Kit, using a piece of foot tissue. A partial region of the mt cytochrome c oxidase I gene (COI) was sequenced in all 373 specimens. From a subset of 54 individuals (including representatives of the major mt clades, type localities, peripheral geographic regions and contact zones of distinct mt clades), two additional markers were amplified: a section of the mt 16S gene, as well as a nc sequence comprising almost the entire sequences of the histone 4 and 3 genes and the complete internal spacer region (H4/H3). The two histone genes are orientated in opposite direction and are separated by a non-coding spacer region, an arrangement which is probably not universal in gastropods but was verified so far particularly for species of the informal group of Orthurethra (sensu [25]). The COI and 16S sequences were also amplified in the eleven outgroup specimens. The COI forward primer *COIfolmerFwd* [14] is a variant of the standard primer *LCO1490* [26]; *H2198-Alb* [10] was used as reverse primer. New 16S primers were designed for the amplification of a fragment of approximately 850 bp. The forward primers, *16SLOrc1_fw* and *16SLOrc2_fw*, bind about 50 bp away from the 5′-end of the *16S rRNA* gene and the reverse primer *16SLOrc_rv* is situated in a conserved region about 850 bp downstream. The H4/H3 primers, *OrcH4_left1* and *OrcH4_left2* (positioned at the 3′-end of the H4 gene) were designed based on alignments of H3 and H4 sequences from GenBank. The reverse primer *OrcH3_right1* (at the 3′-end of the H3 gene) was published by [27]. Internal primers for sequencing (*OrcH3S_right3* and *OrcH4S_left3*) were positioned close to the spacer in the coding sequences to obtain the complete 1100 bp fragment with two sequencing runs. The PCR primers (*OrcH4_left1*, *OrcH4_left2* and *OrcH3_right1*) cover a wider spectrum of Orthurethra taxa, while the internal primers (*OrcH3S_right3* and *OrcH4S_left3*) were especially adapted to the genus *Orcula*. All primers are listed in Table 2.

**Table 2:** Primer sets for amplification and sequencing.

Region	Primer (5′ to 3′)	Origin	Fragment size	T Phusion	T Roche
COI fwd	*COIfolmer_fwd:* *GGTCAACAATCATAAAGATATTGG*	[26]	655 bp	54°C	50°C
COI rev	*H2198-Alb:* *TATACTTCAGGATGACCAAAAAATC*	[10]			
16S fwd	*16SLOrc1_fwd:* *TTACCTTTTGCATAATGGTTAAACTA*	presentstudy	c. 850 bp	60°C	54°C
	*16SLOrc2_fwd:* *TTACCTTTTGCATAATGGTTAAATTA*	presentstudy			
16S rev	*16SLOrc_rev:* *CGGTCTGAACTCAGATCATG*	presentstudy			
H4/H3 fwd	*OrcH4_left1:* *GTGCGTCCCTGGCGCTTCA*	presentstudy	c.1100 bp	71°C	57°C
	*OrcH4_left2:* *GGCGCTTCAGGGCGTACACT*	presentstudy			
H4/H3 rev	*OrcH3_right1:* *TGGGCATGATGGTGACACGCT*	[27]			
intern fwd	*OrcH4S_left3:* *CGGTCTGAACTCAGATCATG*	presentstudy			
intern rev	*OrcH3S_right3:* *CGGTCTGAACTCAGATCATG*	presentstudy			

The annealing temperatures are provided for the Finnzymes Phusion and the RocheTaq Polymerase, respectively.

### PCR Amplification and Cloning

COI and 16S fragments were amplified with the Roche Taq DNA polymerase for direct sequencing. The PCR started with a denaturation step for 3 min at 94°C, followed by 35 cycles with 30 s at 94°C, 30 s at the particular annealing temperature (Table 2), and 1 min at 72°C, followed by a final extension for 7 min at 72°C. The PCR for the nc H4/H3 fragments was performed with the standard protocol of the Finnzymes Phusion polymerase, which has proofreading activity. PCR started with a denaturation step for 30 s at 98°C, followed by 35 cycles with 10 s at 98°C, 10 s at the particular annealing temperature (see Table 2), and 30 s at 72°C, followed by a final extension for 7 min at 72°C. Purification and sequencing of the PCR products (both directions) were performed at LGC Genomics (Berlin, Germany), using the PCR primers for sequencing, except for the H4/H3 section, which was sequenced with the internal primers *OrcH3S_right3* and *OrcH4S_left3*. The cloning of PCR products was performed for the H4/H3 primer design phase and for those individuals that proved to be heterozygous with respect to insertions and deletions (indels), resulting in varying spacer lengths and thus impeding direct sequencing. Nine of the 14 specimens with heterozygous sequences yielded fragments differing in spacer length. The fragments were purified using the QIAquick Gel Extraction Kit (QIAGEN), extended by A-endings with the DyNAzyme II DNA polymerase (Finnzymes) and cloned with the TOPO TA cloning kit (Invitrogen). Two to four clones were sequenced until the two main variants were obtained. In two cases (samples WIW2_392 and MLF1_3939) more than two length variants were obtained, which is not completely unexpected in multi-copy gene families. Sequencing was also performed at LGC Genomics (Berlin) using M13 universal primers. All sequences are deposited in GenBank under the following accession numbers: KC568830–KC569204, KJ656162–KJ656172 (COI); KC569205–KC569260, KJ656173–KJ656183 (16S); KC569261–KC569327 (H4/H3).

### Sequence Analysis and Phylogenetic Tree Calculation

Sequences were edited using Bioedit 7.1.3 [28]. When directly sequenced H4/H3 fragments provided ambiguous positions, the respective sites were filled with the corresponding IUPAC codes. The complete COI data set comprises 374 sequences (including a single specimen of *O. conica* as outgroup). The alignment of the 655 nucleotide sites was straightforward because there were no indels. Statistical analyses were performed using all COI sequences. Haplotype and nucleotide diversity were calculated with DnaSP 5.10 [29], and uncorrected genetic *p*-distances between the clades of *O. dolium* and *O. conica* were calculated with MEGA 5.2 [30]. For phylogenetic tree calculation, identical haplotypes from the same geographic areas (SOIUSA codes) were collapsed resulting in a total of 220 COI sequences of *O. dolium* (197 haplotypes). Prior to the phylogenetic tree inference, a search for the best fitting substitution model was performed with jModelTest 0.1 [31]. A Bayesian inference (BI) was calculated with MrBayes 3.2.2 [32], [33] for 5×10^6^ generations (samplefreq = 100; nruns = 2; nchains = 4), applying the parameters obtained from the model test (GTR+G+I; nst = 6, rates = invgamma). Tracer 1.5 [34] was used to assess whether the two runs had converged and when the stationary phase was reached. The first 25% of the trees were discarded as burnin and a 50% majority rule consensus tree was calculated from the remaining trees.

The 16S sequences (55 specimens including *O. conica*) were aligned with ClustalX [35] using default parameters. The original alignment contained 879 positions of which all 61 gap positions were excluded with TrimAl 1.3 [36], implemented in the Phylemon 2.0 web tools [37], using the “no gap” option. Another 84 sites were excluded by performing the “strict” option, leaving 734 positions in the final alignment for the phylogenetic tree analyses. Of those, 217 sites were variable, compared to 331 in the original and 298 sites in the “gaps excluded” alignment. The “no gap” alignment was also used for calculation of uncorrected *p*-distances. The BI was performed with two data partitions (COI and 16S “strict”), using the substitution models suggested by jModelTest 0.1 [31] (COI: HKY+I+G: nst = 2, rates = invgamma; 16S “strict”: HKY+I+G: nst = 2, rates = invgamma), and allowing MrBayes to evaluate the model priors independently. A Maximum Likelihood (ML) tree was calculated with MEGA 5.2 [30], applying the sequence evolution model GTR+G (5 rate categories)+I and performing 1000 bootstrap replicates with SPR (Subtree-Pruning-Redrafting) as heuristic method for tree inference. Support values of the ML analysis are provided for the combined COI and 16S tree.

A Median-Joining network was calculated from the H4/H3 data with Network 4.6.0.0 (Fluxus Technology Ltd.) using the default settings. Two networks were calculated: one without gaps and one keeping the gap positions. The networks were then post-processed to reduce unnecessary median vectors using the MP option. As the gap positions contained valuable information, we show the network including the gap sites.

### Molecular Clock Analysis and Geographic Range Reconstruction

A reconstruction of the historical biogeography of *O. dolium* was performed with Lagrange 2.0.1 [38]. Lagrange 2.0.1 uses a dispersal-extinction-cladogenesis (DEC) modeling, which allows analyzing the ML values of rate transitions as a function of time. The calculations were based on a molecular clock dated linearized BI tree calculated with BEAST 1.7.5 [39]. The fossil record of *O. dolium* comprises only material of the Holocene and the Middle and Late Pleistocene. This might be due to the fact that during these time periods climate conditions promoted the accumulation of loess sediments containing high numbers of gastropod shells. In contrast, fossil records of the Early Pleistocene and the Pliocene are very scarce and lack many land snail species endemic to Central Europe, among them *O. dolium*. Consequently, dating the stem of the tree with the species first occurrence in the fossil record was not a reasonable option. Hence, substitution rates were inferred from a comparison of two other Orculidae, *Orculella aragonica* and *O. bulgarica*. The latter is widespread in South-Eastern Europe and Western Asia, whereas its sister species, *O. aragonica*, is distributed only in the Iberian Peninsula [22]. The earliest fossil record of *O. aragonica*/*O. bulgarica* dates from about 1.8 mya at the Almenara-Casablanca karst complex (Castellón, Eastern Spain), which contains Miocene to Early Pleistocene sediments [40], [41]. We assumed that the earliest record coincides with the time period when the ancestral lineages of the two species split from each other. Calculation of substitution rates and molecular clock analysis were both performed with the same COI and 16S (“strict”, 696 positions) alignments (including the sequences of *O. conica* and the two *Orculella* species). For each partition, TN93+G was applied both to the measurement of substitution rates and the inference of the molecular clock dated tree. Molecular clock tests were performed independently for the COI and 16S alignments with MEGA 5.2 under the TN93 model, using a discrete Gamma (G) distribution to model differences in evolutionary rates among sites. The null hypotheses of equal evolutionary rates throughout the trees were rejected at a 5% significance level. Substitution rates (COI: 0.02333, 16S: 0.02528; substitutions/ma) were assigned in the prior settings of BEAUti 1.7.5 (part of the BEAST package), and uncorrelated relaxed lognormal molecular clocks were implemented for both sequence partitions. “Speciation: Yule Process” was chosen as tree prior. The BEAST analysis was performed with four independent runs with 10^7^ generations each (sample freq.: 1000). Tracer 1.5 [34] was used to assess whether the four runs had converged. The four independent runs were then combined with LogCombiner 1.7.5 (part of the BEAST package). Subsequently, 25% of the trees were discarded as burnin and a 50% majority rule consensus tree was calculated from the remaining 3×10^5^ trees. Median node heights and 95% highest posterior density (95% HPD) intervals are provided for major nodes in the results section. The rate-calibrated linearized tree was then prepared for Lagrange configurator 20130526, together with a range matrix in which each lineage was assigned either to the Northern Calcareous Alps, the Southern Calcareous Alps, the Western Alps or the Western Carpathians. Migration was permitted between all regions, but lower probabilities (‘0.5’ instead of ‘1.0’) were assigned in the dispersal constraints for migration between areas not being immediately adjacent (Southern Calcareous Alps and Western Carpathians; Southern Calcareous Alps and Western Alps; Western Carpathians and Western Alps). The ancestors were allowed to occupy a maximum of two geographic areas. Alternative analyses were run with unlimited range sizes (allowing the taxa to inhabit all four geographic areas) and assigning the same probabilities (‘1.0’) for migration between the four areas in the dispersal constraints.

### Literature Search for Fossil Records of *O*. *dolium*

We screened various papers for fossil records of *O. dolium*. The dating of the Alpine sites seems to be rather tentative because all but one site were dated only using reconciliation with vertebrate fossils of the same layers. Most reliable is the stratigraphic dating of recently investigated loess profiles of the Pannonian Basin, including measurements of carbonate content variations, low-field magnetic susceptibility and radiocarbon dating using macro charcoal fragments. Most of the Western Carpathian records are based on investigations of soil profiles as well. The publications with positive records of *O. dolium* are listed according to the countries investigated: Austria [42], [43], Croatia [44], [45], Czech Republic [46]–[48], Germany [49], Hungary [19], [50], Serbia [51]–[53] and Slovakia [47], [48]. Since the evaluation of the fossil record of *Orcula dolium* was based on literature data only, no permits were required for that part of the study. We assigned each record to a time period of either cold or warm climate phases of Middle and Late Pleistocene according to the Quaternary divisions of the North European climate cycles of Zagwijn [54]: the Weichselian (115–11 ka ago), Saalian (350–130 ka ago) and Elsterian (475–370 ka ago) were considered as cold climate stages (glacials), compared to the warm stages (interglacials), Holocene (11 ka ago - present), Weichselian-Saalian interglacial ( = Eemian; 130–115 ka ago) and Saalian-Elsterian interglacial ( = Holsteinian; 370–350 ka ago). However, it has to be noted that massive glaciations of the Alps and northern Europe occurred only at some times of the glacials, most recent during the LGM (30–18 kya). The maps showing the distribution of genetic clades and fossil records were prepared using ArcMap Desktop 10.0 and manually edited in Adobe Photoshop CS4 version 12.

## Results

### Mitochondrial Clades

**Figure 2:**
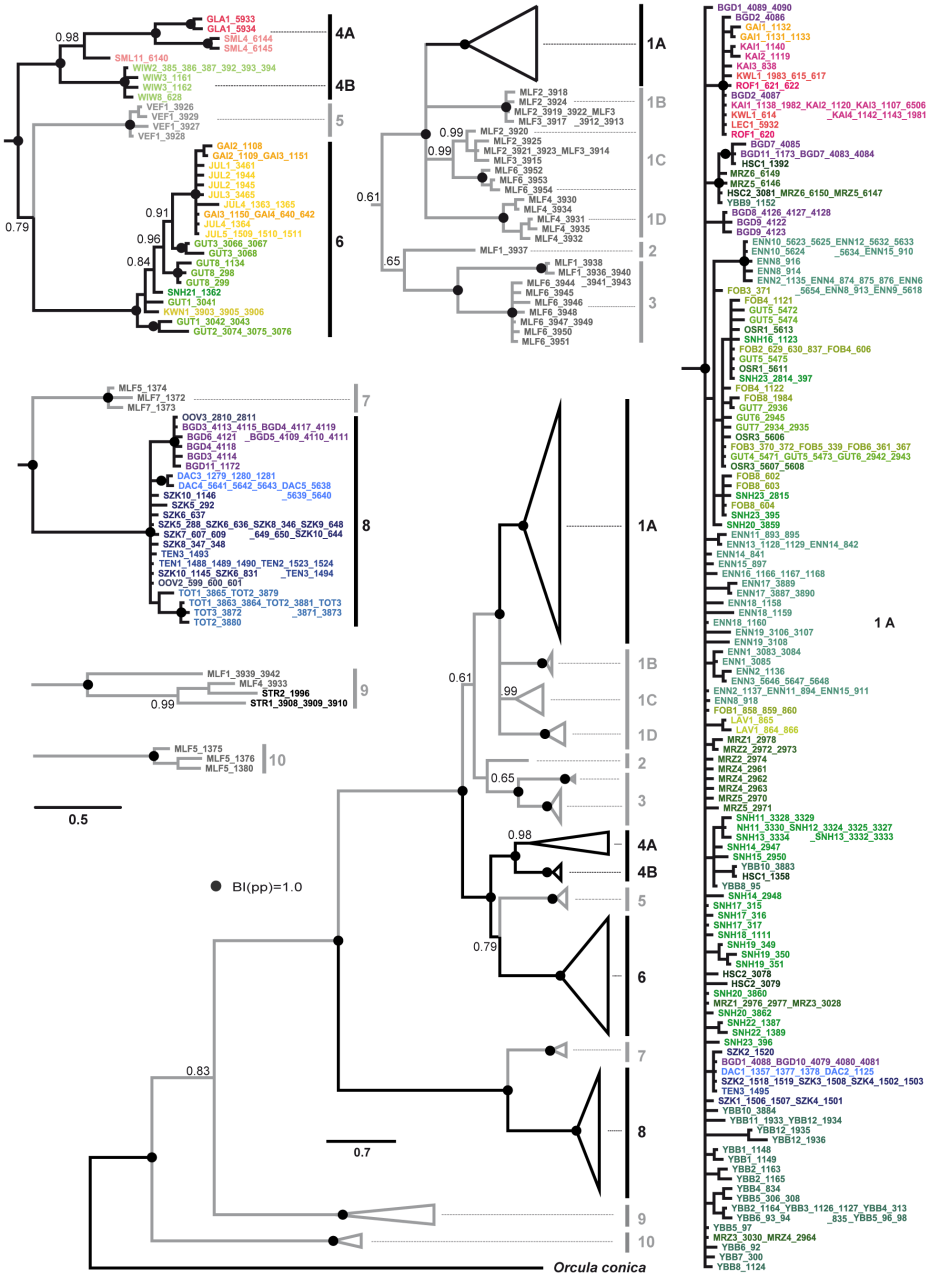
BI analysis of COI haplotypes. The central tree provides an overview. Posterior probabilities are given at the main nodes. Black dots indicate nodes with maximum support. A specimen of *O. conica* is used as outgroup. More details are provided in the partial trees with different colors corresponding to the geographic mountain regions depicted in Fig. 1. The scale bars indicate the expected number of substitutions per site according to the model of sequence evolution applied.

**Figure 3:**
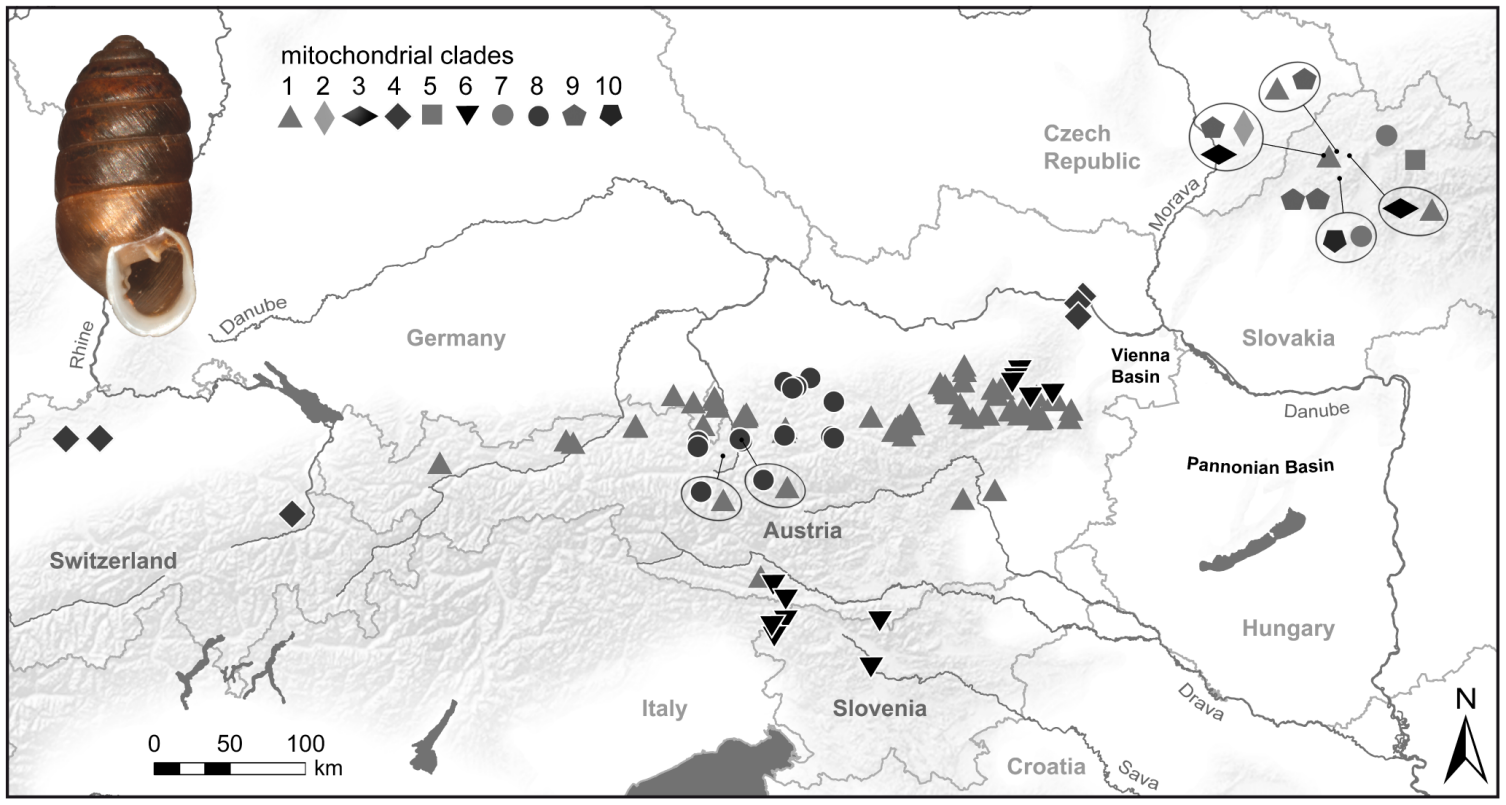
Distribution of localities investigated in the Alps and the Western Carpathians. The symbols indicate the presence of the respective mt clades. If specimens of different mt clades are present at the same localities, the respective clade symbols are shown encircled with a line pointing towards the locality. A picture of the shell of a type specimen of *O. dolium* (Syntype NHMW 14765/1820.26.61/2; size: 6.8 mm in height) is provided in the left upper corner.

**Figure 4:**
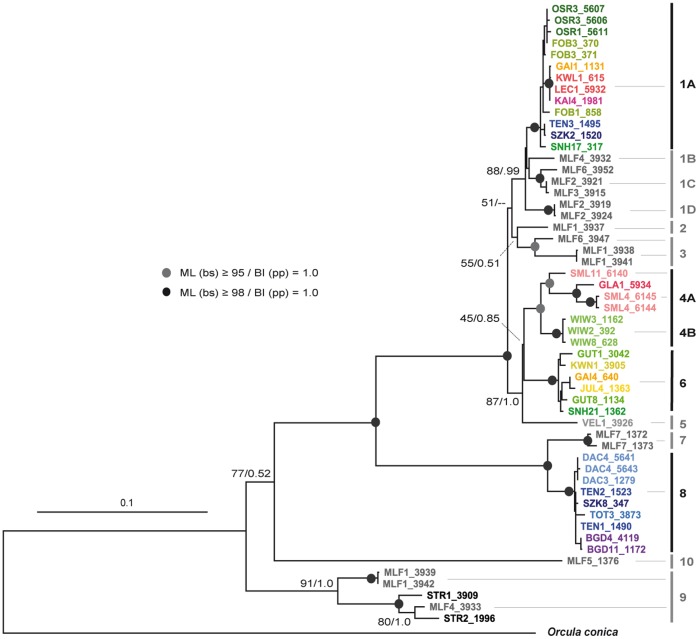
BI tree of the concatenated COI and 16S sequences. Posterior probabilities and ML bootstrap values are provided for all nodes above sub-clade level. The scale bar indicates the expected number of substitutions per site according to the model of sequence evolution applied. The dots indicate nodes with posterior probabilities of 1.0 and bootstrap values of more than 98% (black) or 95% to 97% (grey) in the ML analyses, respectively.

Among the 373 individuals of *O. dolium* investigated, 197 COI haplotypes are observed. The phylogenetic trees calculated with different algorithms show similar topologies. Ten major clades, some of them divided into distinct sub-clades, are highly supported in all analyses. Six of the major clades occur exclusively in the Western Carpathians (2, 3, 5, 7, 9 and 10), three clades are distributed solely in the Alpine region (4, 6 and 8). The final clade, clade 1, is subdivided into four sub-clades themselves restricted to particular regions (Alps: 1A; Western Carpathians: 1B, 1C and 1D) (Figs. 2, 3 and 4). Sub-clade 1A is distributed throughout the Northern Calcareous Alps but occurs also in a distinct area of the Southern Calcareous Alps. Similarly, clade 6 is distributed in separate areas of the Northern Calcareous Alps and the Southern Calcareous Alps. Clade 4 also has a disjunct distribution, but in an east-west orientation, occurring in the eastern-most part of the Northern Calcareous Alps (4B) and in the Western Alps (4A). In contrast, clade 8 is geographically restricted to a small area in the Northern Calcareous Alps. The BI tree based on COI and 16S reveals the same highly supported clades, sub-clades and overall topology as the COI tree, but some nodes are better supported. Nevertheless, the topology remains partly ambiguous, e.g., the relationships between clades 1, 2 and 3 as well as between 4, 5, and 6 are unresolved trichotomies.

### Nuclear Data Set

**Figure 5:**
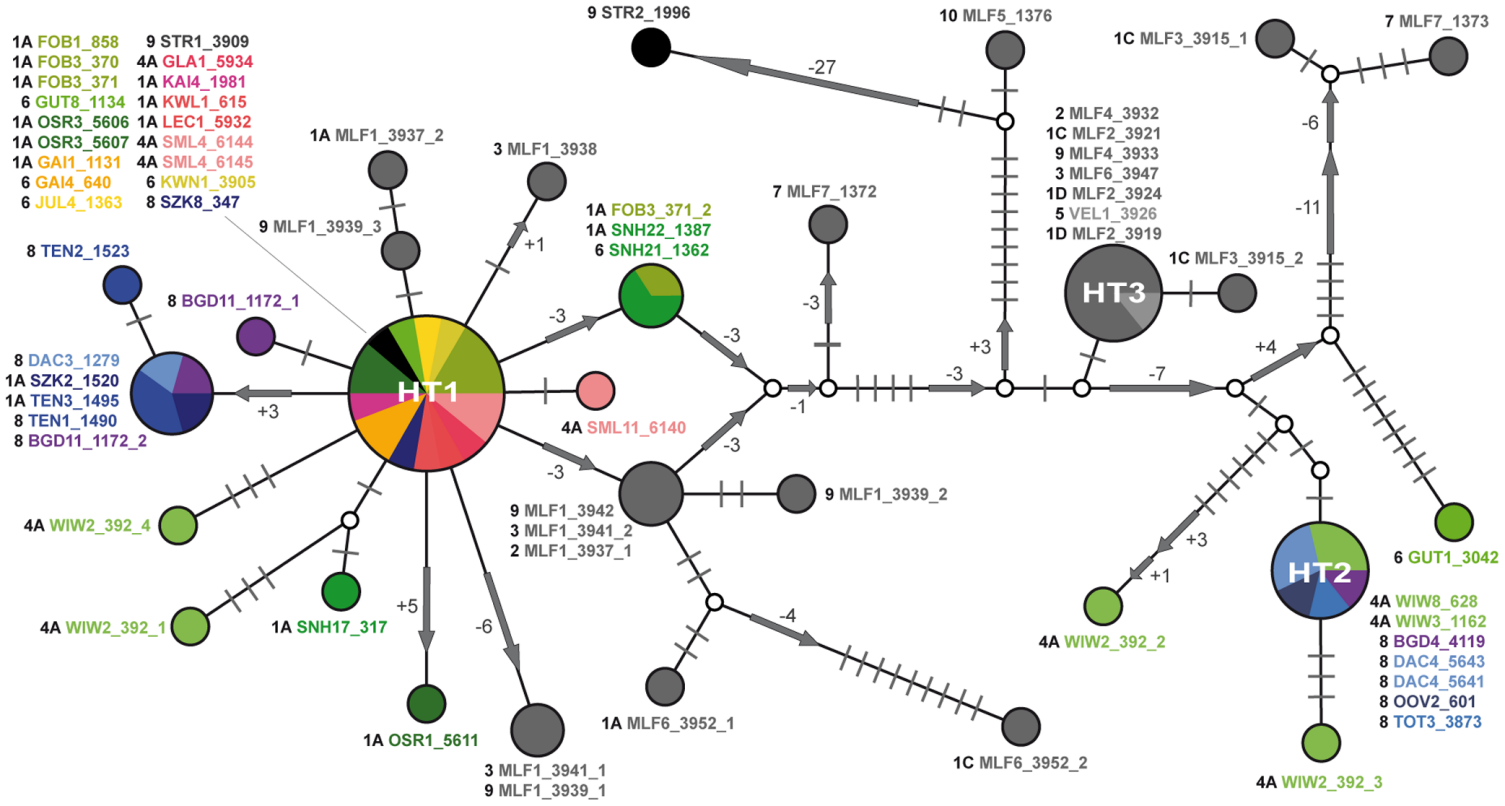
Median Joining network of the H4/H3 sequences. Bars indicate substitutions within the H3, H4 and the spacer region. The loss or gain of bases (indels) in the spacer region is displayed by arrows pointing in the respective direction and the numbers indicate in how many bases the haplotypes/alleles differ from each other. The three most common alleles are named as HT1, HT2 and HT3. The size of the circles corresponds to the number of sequences providing the same allele. The colors correspond to one of the mountain regions defined in Fig. 1. To facilitate the comparison of nc and mt data, the specimen labels and the clade affiliation are indicated next to the haplotype circles.

The H4/H3 data is shown as a phylogenetic network (Fig. 5). It is not possible to incorporate the division of the mt clades into the H4/H3 network because the pattern differs considerably from that obtained in the mt trees - specimens of the same mt clades often provide very different H4/H3 alleles. The general scheme of the network exhibits three frequent alleles (HT1, HT2 and HT3), each encircled by several similar haplotypes, which differ by a few substitutions or indels only. Additionally, there are several unique alleles, particularly in specimens from the Western Carpathians (e.g. in 1996_STR2, 1376_MLF5, 3952_MLF6, 3915_MLF3 and 1373_MLF7). In the Alpine populations, HT1 is the most frequent allele and, apart from a unique Western Alpine allele (differing by 2 substitutions from HT1), it is the only one found in the Western Alps and the Southern Calcareous Alps. In the Western Carpathians, HT1 is detected in a single specimen (STR1_3909) only. However, HT1 is encircled by several slightly differing haplotypes of both the Northern Calcareous Alps and the Western Carpathians. In contrast, HT2 and HT3 are each found exclusively in either the Northern Calcareous Alps or the Western Carpathians, respectively. Thus, all populations having HT2 additionally feature HT1 or haplotypes slightly deviating from HT1. Within the Alps, the populations of the eastern-most margin of the Northern Calcareous Alps exhibit the largest allele diversity.

### Genetic Diversity

Intraspecific distances are extraordinarily high for the COI gene, with up to 18.4% mean *p*-distance between the clades (Table 3). The maximum *p*-distance within the Alpine populations is 16.9%, compared to 18.3% in the Western Carpathian ones. The mean *p*-distance between *O. dolium* and *O. conica* is only slightly higher at 18.4% (Table 3). The respective haplotype and nucleotide diversities of the COI clades are high in all populations (Table 4). Distances were also calculated for the 16S sequences (Table 5). The largest mean *p*-distance between clades of *O. dolium* is 14.4%, the distance between *O. dolium* and *O. conica* is 19.3%, indicating that the conserved parts of the 16S suffered less from saturation than COI. Regarding the nc H4/H3 sequences, the Western Carpathian populations almost consistently show larger *p*-distances (max.: H3 1.5%, H4 2.7%, Spacer 2.6%; clones excluded: H3 0.3%, H4 1.6%, Spacer 1.0%) than the Alpine ones (max.: H3 0.6%, H4 1.9%, Spacer 2.1%; clones excluded: H3 0.3%, H4 1.2%, Spacer 1.5%). The max. *p*-distances within *O. dolium* (H3 1.5%, H4 2.7%, Spacer 2.6%; clones excluded: H3 0.3%, H4 1.9%, Spacer 1.8%) are comparable to the mean distances between *O. dolium* and *O. conica* (H3 0.67%, H4 1.30%, Spacer 2.38%).

**Table 3:** Mean and maximum genetic *p*-distances (in %) for the COI sequences.

mt clade	1A	1B	1C	1D	2	3	4A	4B	5	6	7	8	9	10
dist. Ø	0.9	0.1	0.9	0.5	n/c	2.2	2.6	0.2	0.4	1.2	0.8	0.7	4.1	1.3
dist. max.	2.6	0.3	1.8	0.9	n/c	4.3	3.8	0.6	0.8	2.9	1.1	1.4	6.7	1.4
1A														
1B	3.8													
1C	3.0	3.7												
1D	3.6	4.4	3.3											
2	5.1	5.6	4.8	5.8										
3	5.7	5.9	5.3	5.7	5.1									
4A	7.2	7.7	6.9	7.4	6.7	8.1								
4B	6.1	6.7	5.8	7.0	5.7	7.0	4.6							
5	7.2	7.6	6.8	7.5	5.7	7.1	6.5	5.4						
6	7.1	7.1	7.0	7.7	6.9	7.8	7.4	6.3	6.0					
7	13.5	14.3	14.2	13.6	13.6	13.4	13.6	14.5	13.4	14.8				
8	14.5	15.2	15.2	15.0	14.6	13.9	14.7	16.1	14.7	15.8	5.6			
9	15.4	15.8	15.3	15.1	16.0	15.1	15.1	15.2	15.6	15.9	16.7	16.9		
10	16.9	17.5	16.8	16.2	18.1	16.8	17.2	16.8	17.9	18.4	15.9	16.4	14.2	
*O. conica*	18.0	17.9	18.7	18.2	18.8	18.2	18.6	18.6	18.2	19.1	18.7	20.1	16.6	16.5

**Table 4:** Haplotype and nucleotide diversity within clades for the COI sequences.

mt clade	1A	1B	1C	1D	2	3	4A	4B	5	6	7	8	9	10	Alp.	Carp.	total
**sequence no.**	218	7	9	5	1	13	5	9	4	32	3	57	7	3	**321**	**52**	**373**
**haplotype no. (h)**	111	4	7	5	1	9	5	4	4	18	3	19	4	3	**157**	**40**	**197**
**haplotype div. (Hd)**	0.983	0.714	0.917	1.000	0.000	0.910	1.000	0.583	1.000	0.938	1.000	0.882	0.810	1.000	**0.987**	**0.985**	**0.990**
**nucleotide div. (Pi)**	0.009	0.001	0.008	0.016	0.000	0.022	0.026	0.002	0.004	0.012	0.008	0.007	0.041	0.013	**0.062**	**0.092**	**0.069**

**Table 5:** Mean and maximum genetic *p*-distances (in %) for the 16S sequences.

clade	1A	1B	1C	1D	2	3	4A	4B	5	6	7	8	9	10
**dist. max.**	1.1	–	1.6	0.1	–	3.2	3.6	0.3	–	2.4	0.8	1.1	7.0	–
**1A**														
**1B**	2.1													
**1C**	2.3	1.5												
**1D**	2.0	2.1	2.1											
**2**	2.9	3.0	3.1	3.2										
**3**	4.0	3.7	4.1	3.5	3.4									
**4A**	4.5	4.1	4.0	3.8	4.6	5.1								
**4B**	3.8	3.2	3.2	3.0	3.3	3.7	2.7							
**5**	4.5	4.0	4.4	3.9	3.9	4.1	5.2	4.3						
**6**	3.4	3.2	3.5	3.1	3.0	3.7	3.8	2.8	3.8					
**7**	12.2	12.0	12.5	11.9	12.7	12.8	12.7	12.4	13.2	12.7				
**8**	11.6	11.2	11.6	11.1	11.8	11.8	11.8	11.6	12.3	11.9	2.2			
**9**	13.2	12.8	13.1	12.4	13.7	13.7	13.7	13.4	13.5	13.1	13.8	13.1		
**10**	13.6	13.0	13.7	13.2	14.2	14.2	14.4	14.3	14.0	13.4	14.3	14.1	12.3	
***O. conica***	**19.5**	**19.4**	**19.7**	**19.0**	**20.1**	**20.0**	**19.4**	**19.8**	**19.9**	**19.0**	**19.8**	**18.8**	**17.3**	**19.0**

### Molecular Clock Analysis and Reconstruction of the Phylogeographic Range Evolution

**Figure 6:**
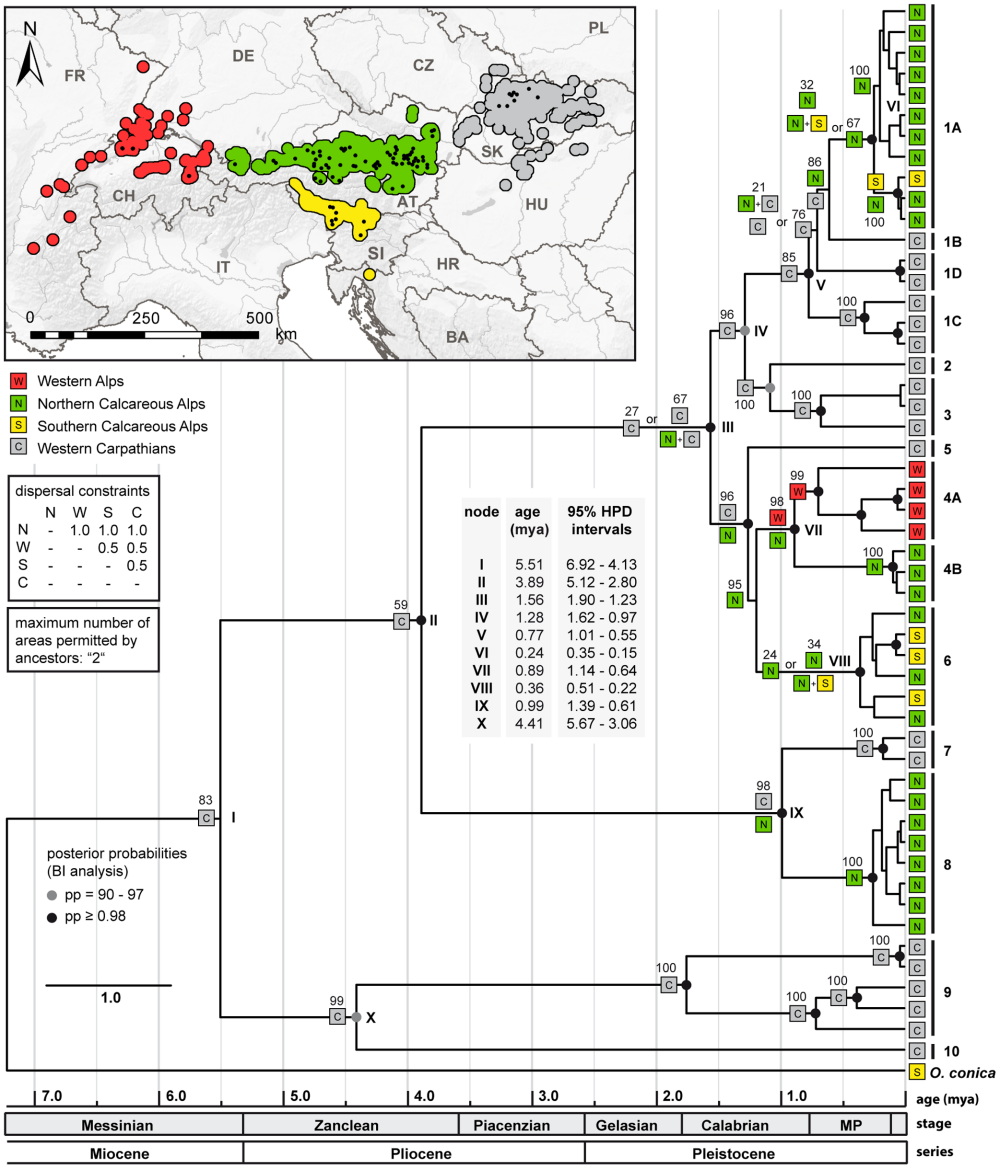
Reconstruction of the geographic range evolution. The map shows the distribution areas of *O. dolium* in the four Alpine and Carpathian mountain areas sampled (encoded by different colors). Small black dots represent localities sampled in the present study. The linearized molecular clock dated BI tree shows the relationships of selected mt lineages (COI/16S data) of *O. dolium*. Black and grey dots indicate nodes with high posterior probabilities (see figure for values). The colored symbols at the branch tips indicate the geographic origin of the haplotypes. The ancestors were allowed to occupy a maximum of two geographic areas. At the cladogenesis events (nodes), all alternative ancestral subdivision/inheritance scenarios with likelihoods of 15% or more are indicated, together with the respective likelihoods, and separated by an “or”. When scenarios for cladogenesis events involve two ancestral areas, the symbol for the likely ancestral area/−s is/are provided left to each of the two branches. For nodes representing major splits, node ages and 95% posterior HPD intervals are indicated. A time scale in mya is given below.

The linearized tree resulting from the BEAST 1.7.5 analysis was combined with a biogeographical range reconstruction using Lagrange v.20130526 (Fig. 6). For the nodes marking major splits of mt lineages, the node ages and the 95% HPD intervals (in mya) are provided. The alternative ancestral subdivision/inheritance scenarios with likelihoods of 15% or more are also indicated in the tree. As a main result, the analyses suggest that *O. dolium* originated 6.92 to 4.13 mya (95% HPD interval) around the Miocene-Pliocene boundary. However, the analyses support that the broad diversification into numerous lineages happened during the Pleistocene. According to the reconstruction of the species’ geographic range history (ancestors allowed to occupy a maximum of two geographic areas/dispersal constraint for areas not immediately adjacent: ‘0.5’), the ancestral *O. dolium* was distributed in the Western Carpathians (Maximum Likelihood of ancestral stage at cladogenesis event: 0.83). The alternative analysis run without range restrictions (ancestors allowed to occupy all four geographic areas/dispersal constraint for areas not immediately adjacent: ‘0.5’) predicted similar ancestral ranges but the most likely ancestral range scenarios generally obtained lower likelihood values. Moreover, alternative ancestral ranges predicted for several nodes comprised differing geographic areas (Fig. S2). Analyses run with the same range constraints as in the two previous analyses, but with differing dispersal constraint (migrations between all areas are equally likely) resulted in identical ancestral ranges for almost all nodes, only the ML values differed slightly (by a maximum of 10 percent) (data not shown).

### Middle and Late Pleistocene Distribution Derived from Fossil Record

**Figure 7:**
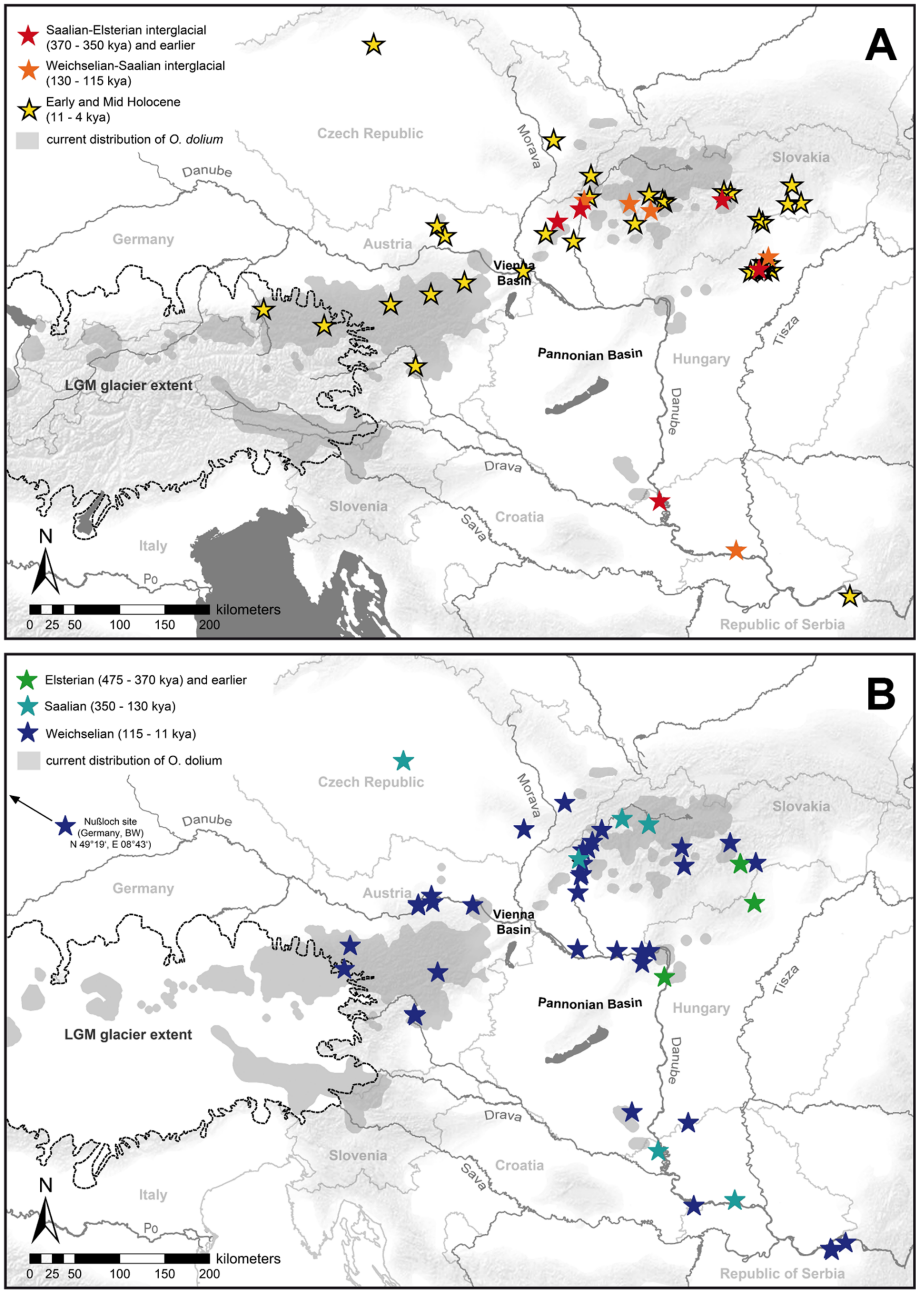
Distribution of fossil *O. dolium* in Central Europe during warm (A) and cold (B) Pleistocene climate stages. The LGM glacier line indicates the maximum extent of glaciers during the Weichselian (35 to 19 ka). The grey-shaded areas correspond to the current distribution of *O. dolium*.

The information about the fossil distribution of *O. dolium* is displayed in Fig. 7. Separate maps are presented for cold and warm Pleistocene climate stages because several localities provide records of both glacial and interglacial periods. The literature reports *O. dolium* from about 100 fossil sites, most of which are located in the Western Carpathians, including 40 sites in Slovakia alone [47], [48]. Four Czech localities provide Middle Pleistocene to Holocene fossils, among them the northern-most confirmed site, which is located about 30 km north of Prague [47]. Around 20 sites are located in the Western Carpathians of Northern Hungary, with Holocene, Weichselian, Eemian and Elsterian deposits [19], [48], [50]. Some of these represent the earliest fossil records of the species, particularly the sites in the Hungarian Bükk mountains and the surroundings of Budapest. Deposits from the Slovenská Skala in South-Eastern Slovakia also date back to the Elsterian and Holsteinian about 400 ka ago [48]. Fewer data are available from Austria and Germany. Most of the 18 sites of the Northern Calcareous Alps are dated to the Holocene or the Weichselian. Only a single Alpine site (Vienna, Austria) was tentatively classified to the end of the Middle Pleistocene by [42]; it is not included in the maps because an assignment to a cold or warm climate stage is not possible. The Nuβloch site (Baden-Württemberg, Germany) is the earliest fossil occurrence in the foreland of the Western Alps in the Early and Middle Weichselian, with an increasing frequency in the Late Weichselian layers [49]. Numerous fossil records of *O. dolium* were reported from loess deposits of the Pannonian Basin [19], [52], [55], most of which are located along the Danube in southern Hungary and Northern Serbia, where the rivers Sava and Danube join. Most Pannonian sites provide Weichselian deposits, although the species was already present close to Novi Sad in Serbia at the end of the Saalian [51]. In Eastern Croatia, fossils of *O. dolium* date back from the Weichselian to the Saalian (over 200 ka ago) [44], [45].

## Discussion

### Origin and Diversification of *O. dolium*

Zimmermann [56] hypothesized that *O. dolium* originated in the Northern Calcareous Alps like several congeneric species. Frank [42] took up the same position and stated that *O. dolium* emerged in the Northern Calcareous Alps in the Early Pleistocene. However, these assumptions regarding the species’ origin are rather tentative because no Pliocene and Early Pleistocene fossils are known from Central Europe. The earliest record of *O. dolium* from South-Eastern Slovakia and North-Western Hungary dates back to the Elsterian (475–370 kya) and even earlier [19], [48], whereas almost all records from Alpine sites were assigned to the Weichselian (115–11 ka ago) or the Holocene; only a single record (Austria, Vienna) was vaguely dated to the Late Middle Pleistocene [42]. In general, the gastropod record before the Middle Pleistocene is extremely scarce because sediments predominantly consist of red clay, being inappropriate for the fossilization of gastropod shells [19]. Besides, environmental dynamics in the mountainous regions, especially in the preferred limestone habitats, offer few opportunities for shell fossilization. Hence, in the present study, hypotheses regarding the origin and diversification of *O. dolium* are mainly based on molecular genetic data.

Contradicting the assumption of Zimmermann [56] and Frank [42], the variability of the mt and nc markers and the geographic distribution of haplotypes support an origin of *O. dolium* in the Western Carpathians. Seven out of ten clades occur in the Western Carpathians, including two highly diverged clades (9 and 10) which split from the basal nodes of the trees. The populations of the Alps and the Western Carpathians are not reciprocally monophyletic - lineages of both areas derive from three nodes in the mt trees each. The number of H4/H3 alleles highly diverged from the three main alleles HT1, HT2 and HT3 is larger in the Western Carpathians as well. The geographic range reconstruction supports a scenario in which the most recent common ancestor was distributed in the Western Carpathians (ML: 0.83) around 6.26 mya (95% HPD: 5.15 to 7.42); the Northern Calcareous Alps were probably settled later (Fig. 6). A scenario in which the MRCA was distributed in both the Western Carpathians and the Northern Calcareous Alps obtained only low support (ML: 0.12). The distributional patterns of mt clades/variants can best be explained by multiple (probably two or three) migrations between Alps and Western Carpathians with single specimens or populations carrying unique or similar mt variants, respectively. Since the Alpine clades are embedded within the diversity of the Western Carpathians, a predominant east-west migration route is most probable. Alternative to scenarios with multiple migrations, the Alpine diversity could have resulted from a single migration involving multiple individuals carrying strongly differing mt variants. However, the geographically distinct distributions of the Alpine mt clades suggest that the lineages evolved independently from each other. Moreover, the molecular clock analysis indicated that the Alpine mt clades separated from their closest related Western Carpathian lineages during different time periods. The results of the analysis suggested that the Alpine mt sub-clade 1A descended from the Western Carpathians rather recently during the Middle Pleistocene, whereas clade 8 and the cluster including clades 4 and 6 separated from their closest related Western Carpathians lineages probably during the Early Pleistocene already. The nc H4/H3 sequence patterns also support at least two independent migration events, as two highly diverged, geographically more or less separated clusters (HT1, HT2 and similar variants) were found.

### Pleistocene Refuges and Postglacial Expansion Routes

One of the present study’s main objectives is the detection of potential glacial refuges of *O. dolium*. The four major limestone areas currently inhabited by *O. dolium* (Western Alps, Northern Calcareous Alps, Southern Calcareous Alps and Western Carpathians) are treated separately, as is the Pannonian Basin, in which the species is apparently not found nowadays.

The extensive fossil record of the Western Carpathians, with data from both glacials and interglacials, confirms that the area was permanently settled, at least during the second half of the Middle Pleistocene and the Late Pleistocene [48]. In particular the extensive record of Weichselian (115–11 ka ago) fossils provides evidence that *O. dolium* was widely distributed during the last glacial (Fig. 7). Moreover, despite the comparably small sample size, the Western Carpathian populations show a large genetic diversity with complex distribution patterns. There is no clear geographic structure regarding the distribution of mt clades and nc alleles, and the data do not indicate a serious loss of genetic diversity due to genetic bottlenecks. Unlike in the Alpine region, the loss of potential habitat presumably was less significant in the Western Carpathians, which were not affected by massive glaciations during Pleistocene cold stages. The scattered distribution of limestone bedrock in the Western Carpathians is another factor, which may have triggered diversification and preservation of numerous genetic lineages.

The eastern part of the Northern Calcareous Alps potentially provided the largest Alpine refuge for calciphilous taxa because it continuously offered non-glaciated limestone areas. Patterns of endemism and comparative phylogeographic analyses in Alpine plants [8] provide additional evidence for refuges in this area. Similarly, the Northern Calcareous Alps harbor a number of endemic species such as *Trochulus oreinos* and *Cylindrus obtusus* which probably originated in that region [14]. Haplotypes of all four Alpine mt clades are found here, with populations located somewhat separated from each other, and therefore suggesting several smaller refuges. Moreover, the respective populations show a high diversity in nc H4/H3 alleles. The most common mt clade (1A) is distributed from Lower Austria (Gutenstein Alps) in the east to Tirol (Lechtal Alps) in the west, spanning a distance of 400 km (Fig. 3). A distinct mt clade (4B) is present at the eastern edge of the Alps (Wienerwald), a region which is geologically somewhat isolated from the Northern Calcareous Alps due to the predominance of siliciclastics in the intermediate region. The large nc diversity with several H4/H3 alleles, each strongly diverged from the most common alleles HT1 and HT2, and the presence of Weichselian fossil deposits support the assumption that the Wienerwald served as a long-term refuge. The Gutenstein Alps at the northeastern margin of the Northern Calcareous Alps may have provided a refuge area for the population exhibiting mt clade 6. Another potential refuge was located close to the former LGM glacier line in Salzburg and Upper Austria - mt clade 8 is distributed exclusively in this region, and fossil deposits indicate the local presence of the species during the Weichselian [42]. Some of the specimens carrying haplotypes of mt clade 8 possess the nc allele HT2 or similar ones, which elsewhere occur in the Wienerwald only. This can either be explained by past gene flow between the two currently separated populations or by ancestral polymorphism, i.e., the persistence of ancestral histone variants in both areas.

The geographic range reconstruction suggests that the Western Alps were settled from the Northern Calcareous Alps during the Middle Pleistocene 1.05 to 0.66 mya (95% HPD interval). Samples from the Western Alpine sites all form a single mt sub-clade (4A) and have the nc haplotype HT1 or similar types. Although material is available from three sample sites only, the Western Alpine populations show higher distances in the mt sequences (max. uncorrected *p*-distance COI: 3.8%) than any other Alpine clade. The unique presence of the highly diverged mt clade 4A provides support for a refuge in the Western Alps. Since the Western Alps were almost completely covered by ice during several glacials, populations may have outlasted the glacial periods in several smaller refuges at the Western Alpine margins of Switzerland and France as was proposed for *Trochulus villosus* [15] or *Carychium tridentatum* [13]. Fossils in Early to Late Weichselian deposits of the Western Alpine foreland (Nuβloch, Baden-Württemberg, Germany) clearly support that assumption, at least for the last glacial period [49]. We have no molecular data from the German and French areas, but a common ancestry of the western populations is supported by similar conchological traits (collection material of the Natural History Museum, Vienna and Naturmuseum Senckenberg, Frankfurt am Main; Harl et al. in prep.).

The Southern Calcareous Alps were almost completely covered by glaciers during the LGM and hence provided only a small potential refuge for calciphilous taxa in the eastern-most part: a small non-glaciated area in the Karawanks [57]. The phylogenetic data argue against a permanent refuge of *O. dolium* in the Southern Calcareous Alps and suggest that clades 1A and 6 probably reached this region rather recently. In the Southern Calcareous Alps, sub-clade 1A is found at a single site (GAI1) only, with haplotypes similar to those in populations of the Tirolian and Bavarian Northern Calcareous Alps. Since both areas were fully glaciated, clade 1A might have crossed the Central Alps in this area after the LGM. Apart from this single locality, all other sites of the Southern Calcareous Alps possessed haplotypes of clade 6, which is distributed in the Northern Calcareous Alps as well. The similarity of clade 6 haplotypes rather indicates a very recent expansion during the Late Pleistocene or Holocene. However, the presence of a distinct variant of clade 6 at a single site in the Karawanks (KWN1) could be an indication for a Southern Calcareous Alpine refuge.

The fossil record indicates a more or less continuous presence of *O. dolium* in the Pannonian Basin at least from the Saalian onwards (over 200 ka ago). The habitats of the Pannonian populations were probably patchily distributed forests near rivers, which were present in the area even during the LGM [7]. The occurrence of trees at the respective sites is additionally supported by the co-occurrence of other woodland species in the same loess strata, such as *Semilimax semilimax*, *Ena montana* or *Aegopinella ressmanni* [45], [58]. One might ask whether riparian drift from Alpine or Western Carpathian regions could account for the presence of *O. dolium* in the fossil record of the southern part of the Pannonian Basin. However, the high abundance of fossil *O. dolium* in the Pannonian Basin indicates a local source. The contemporary absence of the species is probably the result of anthropogenically induced loss of suitable habitat. Ložek [48] stated that deforestation and dehydration are probably the reasons why these areas lack several gastropod species which were still widely distributed during the Eemian. Thus, the expansion of agricultural areas is a reasonable explanation for the decline of *O. dolium* populations in the Pannonian Basin during the Holocene.

### Genetic Differentiation and Taxonomic Considerations

The intraspecific distances measured for the mt genes are among the highest found in pulmonate species (uncorrected *p*-distances: COI, 18.4%; 16S, 14.4%). By comparison, the genetically diverse helicid taxa *Theba pisana* and *Arianta arbustorum* show COI distances of 13.6% [59] and 15% [10], respectively. Regarding the non-protein coding 16S, higher intraspecific distances were found in the clausiliid species *Charpentieria itala* with 24.2% [60]. 16S divergence is also high in the bradybaenid *Euhadra quaesita* with 14.1% [61]. Regarding the nc sequences analyzed, the largest *p*-distance measured within the protein coding H4 and H3 sequences is 0.8%. The highest distance observed in the non-protein coding spacer region is 1.8%. [59] reported *p*-distances of 0.5% in the non-coding ITS1 sequences of *Theba pisana*, whereas the intraspecific sequence divergence within *Arion subfuscus*, a species extremely variable in its mtDNA, is only 0.3% for the ITS1 sequence [62].

Considering the large genetic variability found within the populations of *O. dolium*, the question arises whether some of the lineages might even represent distinct species. However, there are no indications of hybridization barriers that would suggest splitting the groups into different species. The data indicate gene flow between clades, as suggested by the fact that the specimens displaying the main histone gene variants each feature haplotypes of very distant mt clades (see Fig. 5). However, whether genetic groups correspond to currently accepted subspecies remains a problematic issue. More than 20 subspecies have been described for *O. dolium*, equally divided between the Alps and the Western Carpathians [20]. Most were characterized by minor differences in shell shape and the formation of the aperture folds. Our study includes specimens from several type localities of Slovakian subspecies, namely of *O. d. titan* Brancsik, 1887, *O. d. brancsikii* Clessin, 1887, *O. d. minima* Brancsik, 1887, and *O. d. cebratica* Westerlund, 1887 (Table 1 and Fig. S1). However, none of the clades can be definitively attributed to one of these subspecies. For instance, populations of the very slender, large-shelled *O. d. brancsikii* share the same mt haplotypes (clade 1C), and the nc haplotype HT3, with compact, small *O. d. minima* morphs. The very large-shelled *O. d. titan* possesses a diverged mt haplotype within clade 9 but shows the most common nc haplotype HT1, which otherwise is found in the Alps only. The Slovakian *O. d. cebratica* (clade 5) clusters with the Alpine mt clades 4 and 6 but displays a distinct nc haplotype (HT3). Despite the extremely high conchological and genetic variability in the Western Carpathians, we could not detect a clear correspondence between genetic haplotype groups and subspecies ranges defined by [17]. The geographically isolated and conchologically aberrant endemic of the Wienerwald, *O. dolium infima*, is genetically differentiated from other populations in the mt trees (sub-clade 4B). In contrast, individuals from the type localities of *O. dolium gracilior* Zimmermann, 1932, *O. dolium pseudogularis* Wagner, 1912, *O. dolium edita* Ehrmann, 1933, and *O. dolium raxae* Gittenberger, 1978, all possess haplotypes of the homogeneous mt clade 1 and exhibit the nc haplotypes HT1 or its derivatives, none of them forming a distinct sub-clade. The latter two taxa, *O. d. edita* and *O. d. raxae*, initially were of special interest for our study because they were reported to occur only at high altitudes [17], [56]. Nonetheless, specimens from the corresponding localities do not differ from the common types of the surrounding lowlands in the markers analyzed. However, for final taxonomic decisions, the most important question is whether the presumed morphological distinctness of the various subspecies can be confirmed by morphometric investigations. Such analyses are under way to quantify morphological differences and to focus on the effect of altitude on shape formation and shell size (Harl et al., in prep.).

## Supporting Information

Figure S1 **Pictures of selected types of** ***O. dolium*** **subspecies.** Specimens collected at the type localities of the respective subspecies were investigated in the present study. The types shown shall rather be considered as examples for the species’ variability than defined discrete entities of morphologically separated populations. Some of the types indeed represent extreme morphs but transitional forms are found in most populations. The pictures were already published by Harl et al. (2011) together with data on all other currently known subspecies. In the following we provide the collection data of the specimens shown: A: *O. d. dolium* (syntype NHMW 14765/1820.26.61/2), B: *O. d. edita* (syntype LML ALT/5319/1), C: *O. d. raxae* (syntype LML ALT/5354/1), D: *O. d. pseudogularis* (syntype NHMW 56158), E: *O. d. gracilior* (syntype LML ALT/5343), F: *O. d. infima* (syntype LML/ALT5353/1), G: *O. d. brancsikii* (syntype ? NHMW J. N. 22075), H: *O. d. titan* (syntype NHMW 68377 (5448)/3), I: *O. d. cebratica* (syntype MNHG Wstld2090), J: *O. d. minima* (syntype 27044/2). Abbreviations for Museums: NHMW (Naturhistorisches Museum, Wien), LML (Oberösterreichisches Landesmuseum, Linz) and MNHG (Naturhistoriska Museum, Göteborg). The scale bar indicates 5 mm.
(TIF)

Figure S2 **Reconstruction of the geographic range evolution.** The map shows the distribution areas of *O. dolium* in the four Alpine and Carpathian mountain areas sampled (encoded by different colors). Small black dots represent localities sampled in the present study. The linearized molecular clock dated BI tree shows the relationships of selected mt lineages (COI/16S data) of *O. dolium*. Black and grey dots indicate nodes with high posterior probabilities (see figure for values). The colored symbols at the branch tips indicate the geographic origin of the haplotypes. The ancestors were allowed to occupy all four geographic areas. At the cladogenesis events (nodes), all alternative ancestral subdivision/inheritance scenarios with likelihoods of 15% or more are indicated, together with the respective likelihoods, and separated by an “or”. When scenarios for cladogenesis events involve two or more ancestral areas, the symbol for the likely ancestral area/−s is/are provided left to each of the two branches. For nodes representing major splits, node ages and 95% posterior HPD intervals are indicated. A time scale in mya is given below.
(TIF)

## References

[1] Hewitt G (2004) Genetic consequences of climatic oscillations in the Quaternary. Philos Trans R Soc London Ser B Biol Sci 359: 183–195. 15101575 10.1098/rstb.2003.1388 PMC1693318

[2] Muttoni G, Carcano C, Garzanti E, Ghielmi M, Piccin A, et al (2003) Onset of major Pleistocene glaciations in the Alps. Geology 31: 989–992.

[3] Ivy-Ochs S, Kerschner H, Reuther A, Preusser F, Heine K, et al (2008) Chronology of the last glacial cycle in the European Alps. J Quat Sci 23: 559–573.

[4] Holdhaus K (1954) Die Spuren der Eiszeit in der Tierwelt Europas. Abhandlungen der Zool Gesellschaft Wien 18: 1–493.

[5] Benke M, Brändle M, Albrecht C, Wilke T (2009) Pleistocene phylogeography and phylogenetic concordance in cold-adapted spring snails (*Bythinella* spp.). Mol Ecol 18: 890–903. 19254305 10.1111/j.1365-294X.2008.04073.x

[6] Sommer RS, Zachos FE (2009) Fossil evidence and phylogeography of temperate species: “glacial refugia” and post-glacial recolonization. J Biogeogr 36: 2013–2020.

[7] Willis KJ, van Andel TH (2004) Trees or no trees? The environments of central and eastern Europe during the Last Glaciation. Quat Sci Rev 23: 2369–2387.

[8] Tribsch A, Schönswetter P (2003) Patterns of endemism and comparative phylogeography confirm palaeoenvironmental evidence for Pleistocene refugia in the Eastern Alps. Taxon 52: 477–497.

[9] Haase M, Bisenberger A (2003) Allozymic differentiation in the land snail *Arianta arbustorum* (Stylommatophora, Helicidae): historical inferences. J Zool Syst Evol Res 41: 175–185.

[10] Gittenberger E, Piel WH, Groenenberg DSJ (2004) The Pleistocene glaciations and the evolutionary history of the polytypic snail species *Arianta arbustorum* (Gastropoda, Pulmonata, Helicidae). Mol Phylogenet Evol 30: 64–73. 15022758 10.1016/s1055-7903(03)00182-9

[11] Haase M, Misof B (2009) Dynamic gastropods: stable shell polymorphism despite gene flow in the land snail *Arianta arbustorum* . J Zool Syst Evol Res 47: 105–114.

[12] Haase M, Esch S, Misof B (2013) Local adaptation, refugial isolation and secondary contact of alpine populations of the land snail *Arianta arbustorum* . J Molluscan Stud 79: 241–248.

[13] Weigand AM, Pfenninger M, Jochum A, Klussmann-Kolb A (2012) Alpine crossroads or origin of genetic diversity? Comparative phylogeography of two sympatric microgastropod species. PLoS One 7: e37089. 22606334 10.1371/journal.pone.0037089 PMC3351404

[14] Duda M, Sattmann H, Haring E, Bartel D, Winkler H, et al (2011) Genetic differentiation and shell morphology of *Trochulus oreinos* (Wagner, 1915) and *T. hispidus* (Linnaeus, 1758) (Pulmonata: Hygromiidae) in the northeastern Alps. J Molluscan Stud 77: 30–40. 25197157 10.1093/mollus/eyq037 PMC4153987

[15] Dépraz A, Hausser J, Pfenninger M, Cordellier M (2008) Postglacial recolonization at a snail’s pace (*Trochulus villosus*): confronting competing refugia hypotheses using model selection. Mol Ecol 17: 2449–2462. 18422928 10.1111/j.1365-294X.2008.03760.x

[16] Provan J, Bennett KD (2008) Phylogeographic insights into cryptic glacial refugia. Trends Ecol Evol 23: 564–571. 18722689 10.1016/j.tree.2008.06.010

[17] Klemm W (1974) Die Verbreitung der rezenten Land-Gehäuse-Schnecken in Österreich. Denkschriften der Österrerreichischen Akad der Wissenschaften (mathematisch-naturwissenschaftliche Klasse) 117: 1–503.

[18] Lisický MJ (1991) Mollusca Slovenska (The Slovak molluscs). Bratislava: VEDA vydavateľstvo Slovenskej akadémie vied.

[19] Fűköh L, Krolopp E, Sümegi P (1995) Quaternary malacostratigraphy in Hungary. Malacol Newsl 1: 1–219.

[20] Harl J, Sattmann H, Schileyko A (2011) Types of the extant taxa of the landsnail genus *Orcula* Held 1837 (Gastropoda: Stylommatophora: Orculidae). Arch für Molluskenkd 140: 175–199.

[21] Kruckenhauser L, Harl J, Sattmann H (2011) Optimized drowning procedures for pulmonate land snails allowing subsequent DNA analysis and anatomical dissections. Ann Naturhist Mus Wien B 112: 173–175.

[22] Arrébola J, Razkin O, Gómez-Moliner B, Páll-Gergely B (2012) Redescription of *Orculella aragonica* (Westerlund 1897), an Iberian species different from *O. bulgarica* (Hesse 1915) (Gastropoda: Pulmonata: Orculidae). North West J Zool 8.

[23] Marazzi S (2005) Atlante Orografico delle Alpi. SOIUSA.

[24] Hovorka D, Méres Š, Ivan P (1993) Pre-Alpine Western Carpathians basement complexes: lithology and geodynamic setting. Mittleilungen der Österreichischen Geol Gesellschaft 86: 33–44.

[25] Armbruster GFJ, Bohme M, Bernhard D, Schlegel M (2005) The H3/H4 histone gene cluster of land snails (Gastropoda: Stylommatophora): ts/tv ratio, GC3 drive and signals in Stylommatophoran phylogeny. J Mollus Stud 71: 339–348.

[26] Folmer O, Black M, Heah W, Lutz R, Vrijenhoek R (1994) DNA primers for amplification of mitochondrial cytochrome C oxidase subunit I from diverse metazoan invertebrates. Mol Mar Biol Biotechnol 3: 294–299. 7881515

[27] Cadahía L, Harl J, Duda M, Sattmann H, Kruckenhauser L, et al. (2014) New data on the phylogeny of Ariantinae (Pulmonata, Helicidae) and the systematic position of *Cylindrus obtusus* based on nuclear and mitochondrial DNA marker sequences. J Zool Syst Evol Res: in press.

[28] Hall TA (1999) BioEdit: a user-friendly biological sequences alignment editor and analysis program for Windows 95/98/NT. Nucleic Acids Symp Ser 41: 95–98.

[29] Librado P, Rozas J (2009) DnaSP v5: a software for comprehensive analysis of DNA polymorphism data. Bioinformatics 25: 1451–1452. 19346325 10.1093/bioinformatics/btp187

[30] Tamura K, Peterson D, Peterson N, Stecher G, Nei M, et al (2011) MEGA5: molecular evolutionary genetics analysis using maximum likelihood, evolutionary distance, and maximum parsimony methods. Mol Biol Evol 10: 2731–2739. 10.1093/molbev/msr121 PMC3203626 21546353

[31] Posada D (2008) jModelTest: phylogenetic model averaging. Mol Biol Evol 25: 1253–1256. 18397919 10.1093/molbev/msn083

[32] Huelsenbeck JP, Ronquist F (2001) MRBAYES: Bayesian inference for phylogeny. Bioinformatics 17: 754–755. 11524383 10.1093/bioinformatics/17.8.754

[33] Ronquist F, Huelsenbeck JP (2003) MrBayes 3: Bayesian phylogenetic inference under mixed models. Bioinformatics 19: 1572–1574. 12912839 10.1093/bioinformatics/btg180

[34] Rambaut A, Drummond AJ (2009) Tracer v1.5. Available: from BEAST Software website: http://tree.bio.ed.ac.uk/software/tracer/.

[35] Larkin MA, Blackshields G (2007) ClustalW and ClustalX version 2. Bioinformatics 23: 2947–2948. 17846036 10.1093/bioinformatics/btm404

[36] Capella-Gutiérrez S, Silla-Martínez JM, Gabaldón T (2009) trimAl: a tool for automated alignment trimming in large-scale phylogenetic analyses. Bioinformatics 25: 1972–1973. 19505945 10.1093/bioinformatics/btp348 PMC2712344

[37] Sánchez R, Serra F, Tárraga J, Medina I, Carbonell J, et al (2011) Phylemon 2.0: a suite of web-tools for molecular evolution, phylogenetics, phylogenomics and hypotheses testing. Nucleic Acids Res 39: W470–W474. 21646336 10.1093/nar/gkr408 PMC3125789

[38] Ree RH, Smith SA (2008) Maximum likelihood inference of geographic range evolution by dispersal, local extinction, and cladogenesis. Syst Biol 57: 4–14. 18253896 10.1080/10635150701883881

[39] Drummond AJ, Rambaut A (2007) BEAST: Bayesian evolutionary analysis by sampling trees. BMC Evol Biol 7: 214. 17996036 10.1186/1471-2148-7-214 PMC2247476

[40] Robles F, Martínez-Ortí A (2009) Moluscos continentales de los alrededores de Molina de Aragón (Guadalajara, España), con notas sobre *Orculella bulgarica* (Hesse, 1915) (Gastropoda, Orculidae). Iberus 27: 99–105.

[41] Agustí J, Santos-Cubedo A, Furió M, De Marfá R, Blain H-A, et al (2011) The late Neogene-early Quaternary small vertebrate succession from the Almenara-Casablanca karst complex (Castellón, Eastern Spain): Chronologic and paleoclimatic context. Quat Int 243: 183–191.

[42] Frank C (2006) Plio-pleistozäne und holozäne Mollusken Österreichs. Vienna.

[43] Frank C, Terhorst B, Damm B, Thiel C, Frechen M, et al (2011) Pleistocene loess deposits and mollusc assemblages in the Eastern Pre-Alps. Quat Sci J 60: 126–136.

[44] Hupuczi J, Molnár D, Galović L, Sümegi P (2010) Preliminary malacological investigation of the loess profile at Šarengrad, Croatia. Cent Eur J Geosci 2: 57–63.

[45] Molnár D, Hupuczi J, Galović L, Sümegi P (2010) Preliminary malacological investigation on the loess profile at Zmajevac, Croatia. Cent Eur J Geosci 2: 52–56.

[46] Ložek V (2001) Molluscan fauna from the loess series of Bohemia and Moravia. Quat Int 76–77: 141–156.

[47] Ložek V (2006) Last Glacial paleoenvironments of the West Carpathians in the light of fossil malacofauna. J Geol Sci 26: 73–84.

[48] Ložek V (1964) Quartärmollusken der Tschechoslowakei. Rozpr Ústředního ústavu Geol 31: 1–374.

[49] Moine O, Rousseau D-D, Antoine P (2005) Terrestrial molluscan records of Weichselian Lower to Middle Pleniglacial climatic changes from the Nussloch loess series (Rhine Valley, Germany): the impact of local factors. Boreas 34: 363–380.

[50] Sümegi P, Gulyás S, Persaits G, Gergely Páll D, Molnár D (2011) The loess-paleosol sequence of Basaharc (Hungary) revisited: Mollusc-based paleoecological results for the Middle and Upper Pleistocene. Quat Int 240: 181–192.

[51] Marković SB, Oches EA, Gaudenyi T, Jovanovic M, Hambach U, et al (2004) Palaeoclimate record in the Late Pleistocene loess-paleosol sequence at Miseluk (Vojvodina, Serbia). Quaternaire 15: 361–368.

[52] Mitrović B (2007) Pleistocene malacofauna of the Požarevac Danube area (NE Serbia). Geološki Anal Balk poluostrva 68: 81–89.

[53] Marković SB, McCoy WB, Oches EA, Savić S, Gaudenyi T, et al (2005) Paleoclimate record in the Upper Pleistocene loess-paleosol sequence at Petrovaradin brickyard (Vojvodina, Serbia). Geol Carpathica 56: 545–552.

[54] Zagwijn WH (1992) The beginning of the ice age in Europe and its major subdivisions. Quat Sci Rev 11: 583–591.

[55] Marković SB, Bokhorst MP, Vandenberghe J, McCoy WD, Oches EA, et al (2008) Late Pleistocene loess-palaeosol sequences in the Vojvodina region, north Serbia. J Quat Sci 23: 73–84.

[56] Zimmermann S (1932) Über die Verbreitung und die Formen des Genus *Orcula* Held in den Ostalpen. Arch für Naturgeschichte 1: 1–57.

[57] Van Husen D (1997) LGM and late-glacial fluctuations in the Eastern Alps. Quat Int 38–39: 109–118.

[58] Sümegi P, Krolopp E (2002) Quatermalacological analyses for modeling of the Upper Weichselian palaeoenvironmental changes in the Carpathian Basin. Quat Int 91: 53–63.

[59] Greve C, Hutterer R, Groh K, Haase M, Misof B (2010) Evolutionary diversification of the genus *Theba* (Gastropoda: Helicidae) in space and time: A land snail conquering islands and continents. Mol Phylogenet Evol 57: 572–584. 20800098 10.1016/j.ympev.2010.08.021

[60] Scheel BM, Hausdorf B (2012) Survival and differentiation of subspecies of the land snail *Charpentieria itala* in mountain refuges in the Southern Alps. Mol Ecol 21: 3794–3808. 22650615 10.1111/j.1365-294X.2012.05649.x

[61] Watanabe Y, Chiba S (2001) High within-population mitochondrial DNA variation due to microvicariance and population mixing in the land snail *Euhadra quaesita* (Pulmonata: Bradybaenidae). Mol Ecol 10: 2635–2645. 11883878 10.1046/j.0962-1083.2001.01388.x

[62] Pinceel J, Jordaens K, Pfenninger M, Backeljau T (2005) Rangewide phylogeography of a terrestrial slug in Europe: evidence for Alpine refugia and rapid colonization after the Pleistocene glaciations. Mol Ecol 14: 1133–1150. 15773941 10.1111/j.1365-294X.2005.02479.x

