# Supplementary figures and images for: In Search of Glacial Refuges of the Land Snail Orcula dolium (Pulmonata, Orculidae) - An Integrative Approach Using DNA Sequence and Fossil Data
Source: PLoS One. 2014 May 7;9(5):e96012. doi: 10.1371/journal.pone.0096012 (PMC4013069; doi:10.1371/journal.pone.0096012)

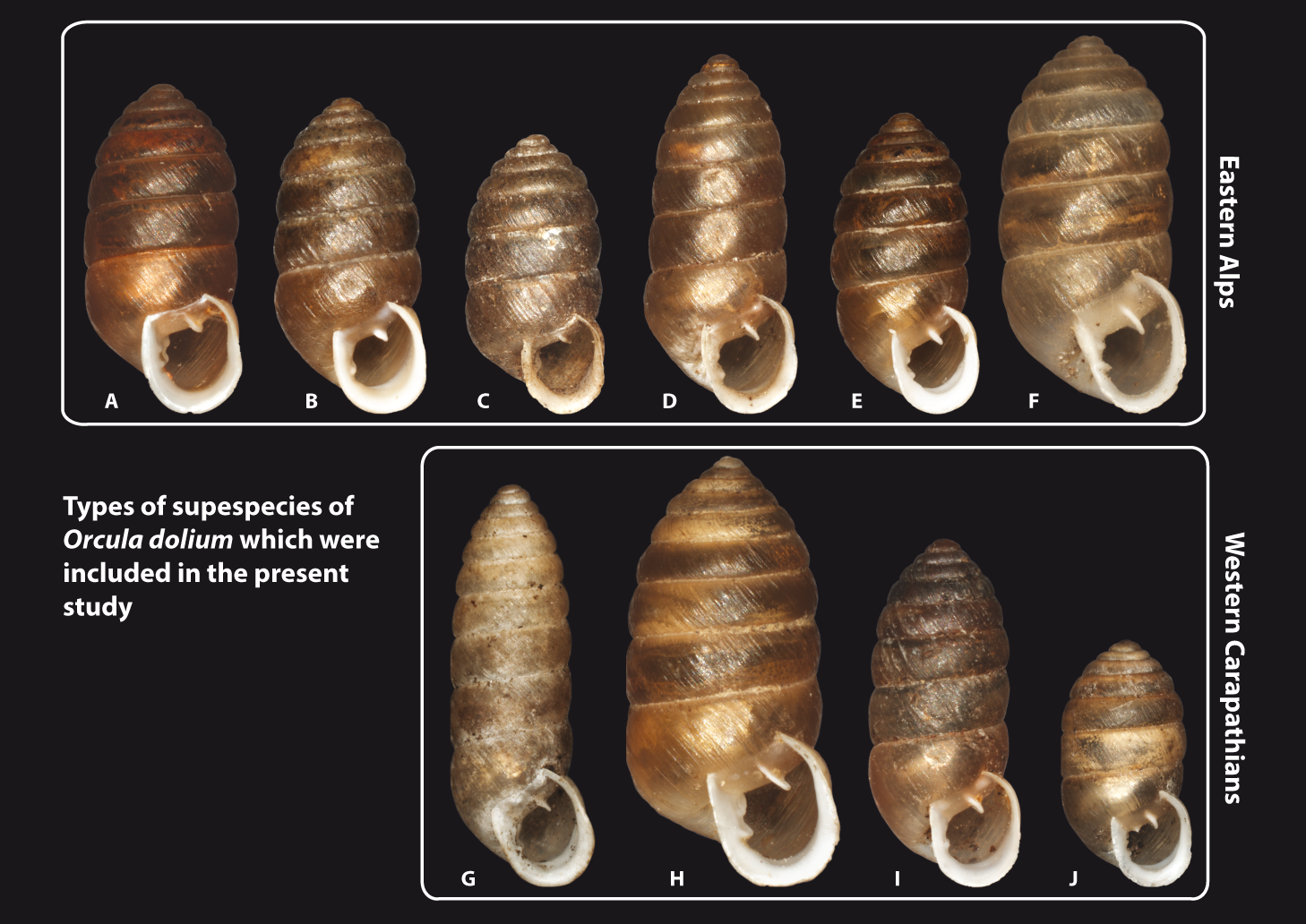

Supplement: Figure S1 — Pictures of selected types of O. dolium subspecies. Specimens collected at the type localities of the respective subspecies were investigated in the present study. The types shown shall rather be considered as examples for the species’ variability than defined discrete entities of morphologically separated populations. Some of the types indeed represent extreme morphs but transitional forms are found in most populations. The pictures were already published by Harl et al. (2011) together with data on all other currently known subspecies. In the following we provide the collection data of the specimens shown: A: O. d. dolium (syntype NHMW 14765/1820.26.61/2), B: O. d. edita (syntype LML ALT/5319/1), C: O. d. raxae (syntype LML ALT/5354/1), D: O. d. pseudogularis (syntype NHMW 56158), E: O. d. gracilior (syntype LML ALT/5343), F: O. d. infima (syntype LML/ALT5353/1), G: O. d. brancsikii (syntype ? NHMW J. N. 22075), H: O. d. titan (syntype NHMW 68377 (5448)/3), I: O. d. cebratica (syntype MNHG Wstld2090), J: O. d. minima (syntype 27044/2). Abbreviations for Museums: NHMW (Naturhistorisches Museum, Wien), LML (Oberösterreichisches Landesmuseum, Linz) and MNHG (Naturhistoriska Museum, Göteborg). The scale bar indicates 5 mm. (TIF) [file pone.0096012.s001.tif]

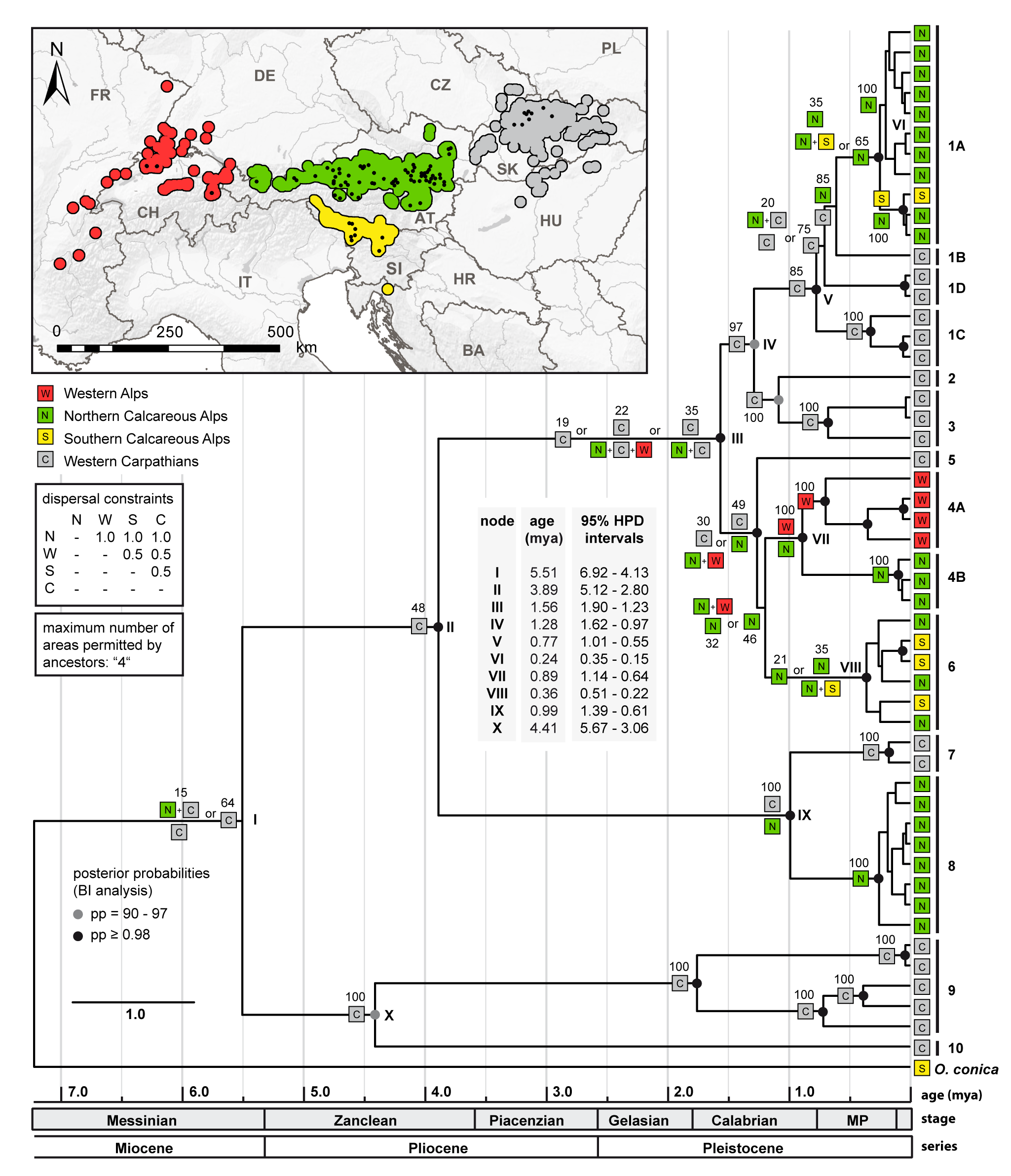

Supplement: Figure S2 — Reconstruction of the geographic range evolution. The map shows the distribution areas of O. dolium in the four Alpine and Carpathian mountain areas sampled (encoded by different colors). Small black dots represent localities sampled in the present study. The linearized molecular clock dated BI tree shows the relationships of selected mt lineages (COI/16S data) of O. dolium. Black and grey dots indicate nodes with high posterior probabilities (see figure for values). The colored symbols at the branch tips indicate the geographic origin of the haplotypes. The ancestors were allowed to occupy all four geographic areas. At the cladogenesis events (nodes), all alternative ancestral subdivision/inheritance scenarios with likelihoods of 15% or more are indicated, together with the respective likelihoods, and separated by an “or”. When scenarios for cladogenesis events involve two or more ancestral areas, the symbol for the likely ancestral area/−s is/are provided left to each of the two branches. For nodes representing major splits, node ages and 95% posterior HPD intervals are indicated. A time scale in mya is given below. (TIF) [file pone.0096012.s002.tif]
